# An optimized bioluminescent substrate for non-invasive imaging in the brain

**DOI:** 10.1038/s41589-023-01265-x

**Published:** 2023-02-09

**Authors:** Yichi Su, Joel R. Walker, Mary P. Hall, Mark A. Klein, Xiang Wu, Lance P. Encell, Kerriann M. Casey, Lan Xiang Liu, Guosong Hong, Michael Z. Lin, Thomas A. Kirkland

**Affiliations:** 1 Department of Neurobiology, Stanford University, Stanford, CA USA; 2 Promega Biosciences, LLC, San Luis Obispo, CA USA; 3 Promega Corporation, Madison, WI USA; 4 Department of Materials Science and Engineering, Stanford University, Stanford, CA USA; 5 Wu Tsai Neurosciences Institute, Stanford University, Stanford, CA USA; 6 Department of Comparative Medicine, Stanford University School of Medicine, Stanford, CA USA; 7 Department of Bioengineering, Stanford University, Stanford, CA USA; 8 Department of Chemical and Systems Biology, Stanford University, Stanford, CA USA

**Keywords:** Imaging, Neuroscience, Chemical tools

## Abstract

Bioluminescence imaging (BLI) allows non-invasive visualization of cells and biochemical events in vivo and thus has become an indispensable technique in biomedical research. However, BLI in the central nervous system remains challenging because luciferases show relatively poor performance in the brain with existing substrates. Here, we report the discovery of a NanoLuc substrate with improved brain performance, cephalofurimazine (CFz). CFz paired with Antares luciferase produces greater than 20-fold more signal from the brain than the standard combination of d-luciferin with firefly luciferase. At standard doses, Antares–CFz matches AkaLuc–AkaLumine/TokeOni in brightness, while occasional higher dosing of CFz can be performed to obtain threefold more signal. CFz should allow the growing number of NanoLuc-based indicators to be applied to the brain with high sensitivity. Using CFz, we achieve video-rate non-invasive imaging of Antares in brains of freely moving mice and demonstrate non-invasive calcium imaging of sensory-evoked activity in genetically defined neurons.

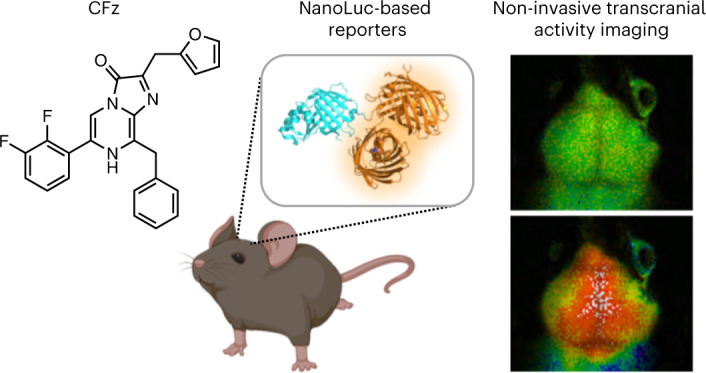

## Abstract (web summary)

Brain imaging using the compact NanoLuc luciferase has been stymied by the lack of suitable substrates. Su et al. report a brain-optimized substrate, cephalofurimazine, and use it to perform non-invasive calcium imaging of neuronal activity.

## Main

Bioluminescence imaging (BLI) is a powerful technique for non-invasive and longitudinal tracking of gene expression, cell location and molecular events in living animals^1–3^. In BLI, a luciferase enzyme, either expressed alone or as a component of a reporter protein, oxidizes a chemical substrate to produce light emission. In contrast to fluorescence imaging, BLI does not require excitation light and is thus free of thickness-dependent autofluorescence and phototoxicity. When using luciferase substrates with negligible spontaneous oxidation, BLI can achieve low to zero background, enabling sensitive non-invasive detection of signals emitted from deep tissues. Direct comparisons have verified the superiority of bioluminescence over fluorescence in signal-to-background ratio for detecting cells through >1 mm of tissue^4^. Thus, over the past two decades, BLI has become routine in mammalian disease models for reporting gene expression and tracking the locations of cells and viruses^3,5^.

However, BLI applications in neuroscience remain uncommon. A major reason is that the blood–brain barrier (BBB) restricts access of traditional luciferase substrates to the central nervous system. For the ATP-dependent insect luciferases, such as the commonly used firefly luciferase (FLuc), their natural substrate d-luciferin was once considered BBB permeable based on its small size and ability to generate some brain signals^6^. However, recent studies revealed that d-luciferin brain delivery is limited. Intraperitoneal (i.p.) injection of d-luciferin produced low signals from the cerebral cortex in *Bdnf-Luc* mice^7^, and d-luciferin is an efficient substrate for the ATP-binding cassette transporter G2 that mediates BBB efflux of small molecules^8^. As a result, researchers have developed synthetic d-luciferin analogs with substantially brighter brain bioluminescence, including CycLuc1 (ref. ^9^) and AkaLumine^10^, also known as TokeOni^7^. In particular, AkaLumine produces more light from the brain than d-luciferin with either FLuc^7^ or the AkaLumine-optimized AkaLuc^10^. The AkaLuc–AkaLumine system allowed video-rate recording of bioluminescence from the brain in freely moving mice and marmosets^10^.

For the ATP-independent marine luciferases, such as *Renilla* luciferase, their natural substrate coelenterazine also suffers from mediocre BBB permeability^11^. Recent marine luciferase engineering efforts led to the development of NanoLuc and its optimized substrate the coelenterazine analog furimazine (Fz)^12^. NanoLuc with Fz exhibits high catalytic activity and thus has found broad applications in biomedical research. For in vivo applications, the blue light emitted by NanoLuc is often red shifted through bioluminescence resonance energy transfer to fluorescent proteins fused to NanoLuc, as exemplified by Antares^13^, Lumifluors^14^ and eNLs^15^. Specifically, Antares, consisting of NanoLuc fused to two CyOFP1 fluorescent protein domains, features an emission peak at 589 nm. In addition, Fz analogs hydrofurimazine (HFz) and fluorofurimazine (FFz) with improved solubility and bioavailability were developed to maximize in vivo brightness from NanoLuc-based reporters^16,17^. Antares with FFz is particularly effective, displaying similar body imaging brightness to AkaLuc with AkaLumine^16,17^. Using these orthogonal BLI systems together enabled simultaneous tracking of tumor size and visualization of chimeric antigen receptor T cells in the same mice^16^. However, while NanoLuc-based reporters have been used for brain imaging^18^, the performance of NanoLuc substrates for brain BLI has yet to be systematically quantified and optimized. Optimizing NanoLuc substrates for use in the brain is particularly important, as NanoLuc is becoming widely used to track the spread of neurotrophic viruses, including severe acute respiratory syndrome coronavirus 2, report on molecular events in neurons and even drive neuronal optogenetics^5,19,20^.

Here, we report the systematic evaluation and optimization of NanoLuc substrates specifically for in-brain applications. Using transgenic mice with Antares expressed in the brain, existing NanoLuc substrates (Fz, HFz and FFz) and new rationally designed structural analogs of Fz were rapidly screened and characterized, resulting in the development of cephalofurimazine (CFz). Antares with CFz at doses compatible with repeated imaging matches the brightness of AkaLuc with AkaLumine, while a higher dose suitable for single use produces 6.5-fold brighter signal. With CFz injected i.p., we achieve video-rate recording of bioluminescence from Antares-expressing neurons in freely moving mice. We also perform a demonstration of non-invasive calcium imaging of sensory-evoked activity in genetically defined neuronal populations in the mammalian brain.

## Results

### Characterizing brain performance of existing NanoLuc substrates

To facilitate comparisons between NanoLuc substrates in mouse brains, we developed transgenic mice with Antares luciferase expressed in specific types of neurons. Specifically, we crossed previously reported mice bearing a CAG*-loxP-*STOP*-loxP-*Antares (CAG-LSL-Antares) transgene in the *H11* safe harbor locus^16^ with tissue-specific Cre recombinase driver mice. Driver lines used in this study included *Camk2a-cre* (forebrain excitatory neurons)^21^, *Vglut2-*IRES*-cre* (excitatory glutamatergic neurons)^22^ and *Vgat-*IRES*-cre* (inhibitory GABAergic neurons)^22^. As the reporters are stably expressed throughout a broad population, mouse-to-mouse variability is lower than with viral transduction, and statistical reliability is increased.

**Fig. 1:**
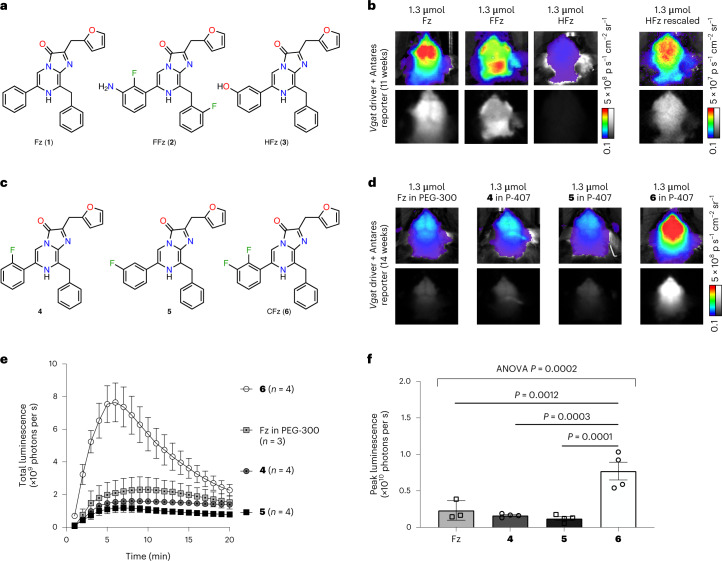
New fluorinated Fz analog with improved brain performance. **a**, Structures of established NanoLuc substrates. **b**, Comparison between NanoLuc substrates in mice doubly hemizygous for *Vgat*-IRES-*cre* and CAG-LSL-Antares transgenes; top, linear pseudocolored; bottom, raw grayscale formats. **c**–**f**, Comparison between Fz and its fluorinated analogs in mice doubly hemizygous for *Vgat*-IRES-*cre* and CAG-LSL-Antares transgenes. Substrates were administered i.p. in a PEG-300-based solution. **c**, Chemical structures of Fz and its fluorinated analogs. **d**, Representative images with peak bioluminescence. **e**, Bioluminescence intensity in the brain over time. Data are presented as mean values ± s.e.m.; *n* = 4 animals for each compound or 3 animals for Fz. **f**, Statistical analysis of peak signal intensities from **e**. *P* values were determined by one-way analysis of variance (ANOVA) followed by Tukey’s post hoc tests. Data are presented as mean values ± s.e.m.

**Extended Data Fig. 1:**
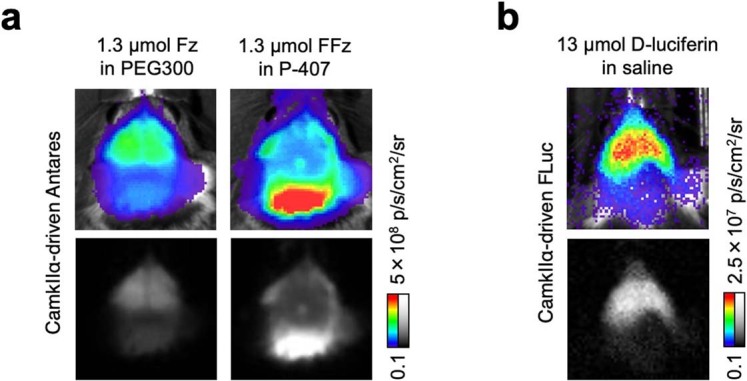
Fluorofurimazine fails to enter the forebrain. **a**, FFz and Fz produce different patterns of light emission in mice doubly hemizygous for *CaMKIIα-cre* and CAG-LSL-Antares. **b**, Light emission after D-luciferin injection in mice doubly hemizygous for *CaMKIIα-cre* and CAG-LSL-FLuc *transgenes*.

We first explored the brightness and signal distribution of existing NanoLuc substrates Fz, HFz and FFz. HFz and FFz (Fig. 1a) are derivatives of Fz with improved aqueous solubility and bioavailability that produce brighter signals for body imaging in mice^16^. In mice doubly hemizygous for CAG-LSL-Antares and *Vgat-*IRES*-cre* transgenes, i.p. administration of Fz produced signal from both the forebrain and hindbrain (Fig. 1b), matching the expected expression pattern for VGAT^21,22^. Unexpectedly, FFz produced a different signal distribution, with low brightness from the forebrain, higher signal from the hindbrain, and highest signal from regions in the head outside the brain (Fig. 1b). We imaged mice with Antares driven by *Camk2a-cre*, which is most active in the forebrain, to test if FFz distribution or reporter expression would dominate in determining signal location. Indeed, while Fz produced the expected forebrain-enriched signal, FFz still produced a signal predominantly from the hindbrain (Extended Data Fig. 1a). As a historic control, d-luciferin with FLuc expressed under *Camk2a-cre* control also showed the expected forebrain-specific signal (Extended Data Fig. 1b). We also tested HFz. HFz produced a signal from the brain similar to Fz, but it was more than tenfold dimmer (Fig. 1b).

### CFz, a substrate with higher brain brightness

The impaired brain performance of HFz and FFz compared to Fz suggested that hydroxy and amino groups may not be ideal for brain applications. To improve brain performance, we explored removing these groups and substituting the same ring, which is the phenyl ring attached to the C6 atom of the bicyclic core, with only fluorine atoms. These derivatives included a 2′-fluoro-substituted compound **4**, a 3′-fluoro-substituted compound **5** and a 2′,3′-difluoro-substituted compound **6** (Fig. 1c). A 4′-fluoro substitution substantially decreased light output, as previously reported^23^, and was not further investigated. Compounds **4**–**6** showed increased solubility over Fz in an aqueous formulation with poloxamer-407 (P-407) as an excipient, a convenient formulation for in vivo studies^24^.

**Extended Data Fig. 2:**
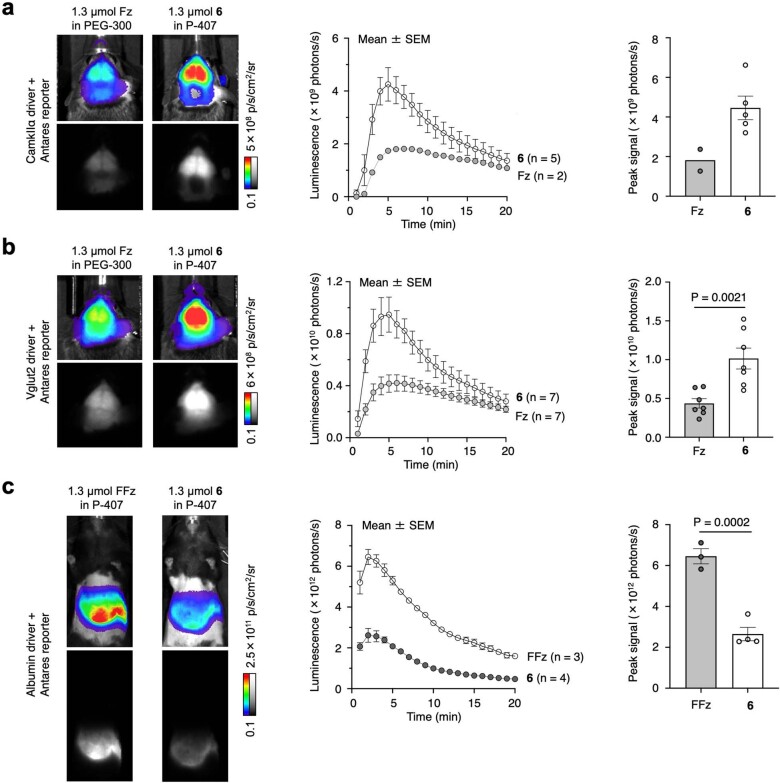
Comparing compound 6 and earlier substrates with additional Cre drivers. Mice doubly hemizygous for CAG-LSL-Antares reporter and a cre driver gene were injected i.p. with 1.3 µmol **6**, Fz, or FFz. In each set: Left, representative images with peak bioluminescence; center, bioluminescence signals over time; right, comparison of peak signals; data are presented as mean values ± SEM. **a**, *CaMKIIα-cre* driver; n = 5 animals for compound **6** and 2 animals for Fz. **b**, *Vglut2*-IRES-*cre* driver; n = 7 animals each for **6** and Fz. Compound **6** was brighter in the brain, with P = 0.0021 by two-tailed Student’s unpaired t-test. **c**, *Alb-cre* driver; n = 4 animals for compound **6** and 3 animals for FFz. FFz was brighter in the liver, with P = 0.0002 by two-tailed Student’s unpaired t-test.

We compared Fz to these new fluorinated analogs **4**–**6** in the mouse brain. We injected 1.3 μmol of Fz (with polyethylene glycol 300 (PEG-300), as 1.3 μmol was not soluble in P-407) or compounds **4**–**6** (with P-407) into CAG-LSL-Antares, *Vgat-*IRES*-cre* double hemizygotes via the i.p. route (Fig. 1d). The new substrates **4**–**6** all produced predominantly cortical signals. While the singly fluorinated substituents **4** and **5** showed similar peak light production as Fz, the doubly fluorinated analog **6** was significantly brighter (Fig. 1e,f). To confirm that the improvement in vivo is not specific to *Vgat*-expressing neurons, we also compared **6** to Fz in mice expressing Antares driven by *Vglut2-*IRES*-cre* or *Camk2a-cre*. In both models, **6** was approximately 2.5-fold brighter than Fz (Extended Data Fig. 2a,b). However, **6** was threefold dimmer than FFz for non-invasive detection of Antares in the liver (Extended Data Fig. 2c).

**Fig. 2:**
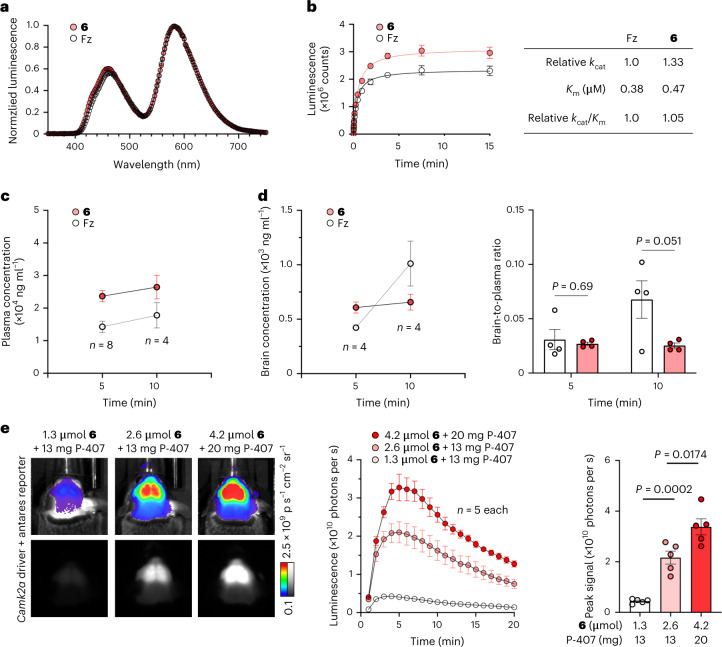
Compound 6 enzymology, PK measurements and formulations. **a**, Emission spectra of Antares when using Fz or compound **6** as substrate. **b**, Determination of kinetic parameters of relative *k*_cat_ and absolute *K*_m_ for Antares with each substrate. Data are presented as mean values *±* s.d.; *n* = 3 technical replicates. Standard deviation was calculated to provide a measurement of the technical reliability of each measurement and not for the purposes of statistical comparison. **c**, Mouse plasma levels of Fz and compound **6** after i.p. administration of 1.3 µmol of either substrate. Data are presented as mean values *±* s.e.m.; *n* = 8 animals for each compound at 5 min and *n* = 4 animals for each compound at 10 min. **d**, Brain concentration (left) and brain-to-plasma ratios (right) of each substrate. Data are presented as mean values *±* s.e.m.; *n* = 4 animals for each compound at each time point. *P* values were determined by two-tailed Student’s unpaired *t*-test. **e**, Dose and vehicle optimization for compound **6** in mice doubly hemizygous for *Camk2a-cre* and CAG-LSL-Antares genes. Representative images at peak bioluminescence (left) and bioluminescence intensity over time (center) are shown. Data are presented as mean values *±* s.e.m.; *n* = 4 animals in each condition. Statistical analysis of peak signals by two-tailed Student’s unpaired *t*-test is shown on the right; *P* = 0.0002 between 1.3 and 2.6 µmol, and *P* = 0.0174 between 2.6 and 4.2 µmol. Compound **6** is hereafter named CFz.

We performed in vitro enzymatic characterization of **6** and Fz. Emission spectra from Antares were similar (Fig. 2a). Relative to Fz, **6** showed a 1.33-fold higher *k*_cat_ and a similar increase in *K*_m_ (Fig. 2b). The fact that **6** improved brain signal by 2.5-fold over Fz, which is disproportionate to the *k*_cat_ improvement, and did so despite a higher *K*_m_ implies that higher free concentrations of **6** are available in luciferase-expressing neurons in vivo.

To study the mechanism behind the improved brain performance of **6**, we performed plasma and brain pharmacokinetic (PK) measurements of **6** and Fz in wild-type mice at 5 and 10 min after substrate injection, time points when their brain signals peaked in Antares-expressing mice. Plasma concentrations indicated that **6** has improved bioavailability after i.p. injection (Fig. 2c). However, the brain concentrations and brain-to-plasma ratios of the two substrates (Fig. 2d) were not well correlated with their brightness in the brain, implying that other factors other than bulk concentrations in brain tissue influence brightness. Specifically, at the time of peak brightness at 5 min, **6** and Fz concentrations reached 1.4 and 1.1 μM, respectively, both above the *K*_m_ values of Antares for these substrates. Thus, a higher effective free concentration of **6**, required to explain its greater light output, may be due to improved transport into neurons (the cell type in which Antares was expressed) that are not discernable in standard PK measurements of total brain tissue. Alternatively, **6** may exhibit less protein or lipid binding so that free concentrations are higher despite similar total tissue concentrations. As the PK measurements were performed in wild-type mice without Antares, consumption of the substrates can be ruled out as a factor.

**Extended Data Fig. 3:**
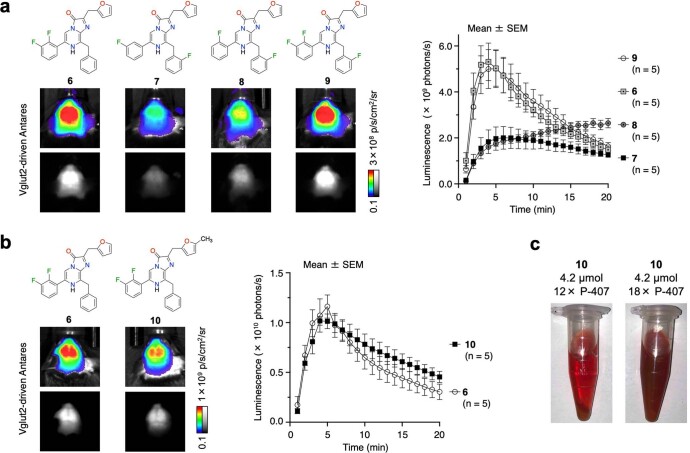
Additional SAR on compound 6. Mice doubly hemizygous for CAG-LSL-Antares reporter and *Vglut2*-IRES-*cre* driver were injected i.p. with 1.3 µmol substrate with 10 mg P-407. **a**, Comparison between **6** and Fz analogues with fluorination on C6 and C8 ring rings. **b**, Comparison between **6** and **10**, a Fz analogue with a methylated furan ring. In **a** and **b**: Left, representative images with peak bioluminescence; right, bioluminescence intensity in the brain over time; data are presented as mean values ± SEM with n = 5 mice in each group. **c**, Representative photos of 4.2 µmol **10** suspended in a formulation with P-407 indicating incomplete solubilization. The experiment was performed twice with similar results.

**Extended Data Fig. 4:**
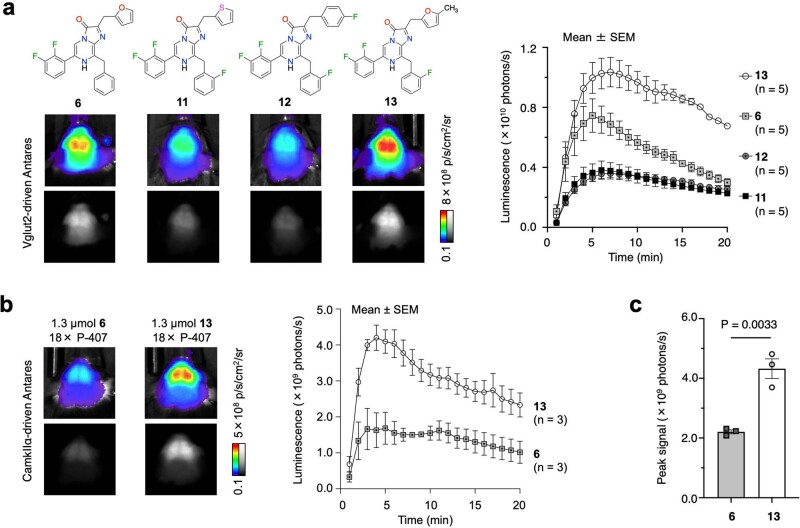
Compound 13 improves brightness. Reporter mice were injected i.p. with 1.3 µmol substrate with 10 mg P-407. **a**, Comparison between **6** and Fz analogues with fluorination on the 2-position of the C8 ring and different groups in place of the furan ring, in mice doubly hemizygous for CAG-LSL-Antares reporter and *Vglut2-IRES-cre* driver; n = 5 animals in each group. **b**, Comparison between **6** and **13**, in mice doubly hemizygous for CAG-LSL-Antares reporter and *CaMKIIα-cre* driver; n = 3 animals in each group. In **a** and **b**: Left, representative images with peak bioluminescence; right, bioluminescence intensity in the brain over time; data are presented as mean values ± SEM. **c**, Comparison of peak signal intensities for the results in **b**. Compound **13** was brighter, with P = 0.0033 by two-tailed Student’s unpaired t-test.

**Extended Data Fig. 5:**
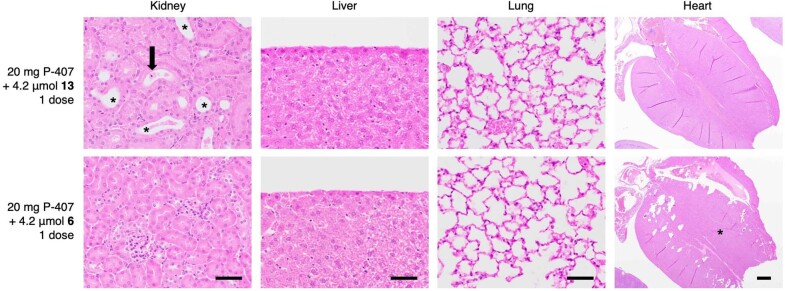
Substrate 13 is more toxic than 6 in vivo. For kidney: asterisks, renal tubular dilation; arrows, renal tubular degeneration and luminal sloughing. For heart: asterisks, myocardial necrosis and hemorrhage. No lesions were noted in the lungs across any groups. The selected images were representative dissection images with the most severe damage of three biological replicates for illustration purposes. Scale bars, 50 μm in kidney, liver, and lung, and 500 μm in heart. Detailed scores of histology analysis of all biological replicates are summarized in Supplementary Table 1.

We performed additional structure–activity relationship analyses to explore whether further improvements were possible beyond compound **6**. Fluorination of **4**–**6** on the C8-attached benzyl ring, found to improve *k*_cat_ previously^16^, did not further improve brain brightness in vivo (Extended Data Fig. 3a). Methylation of **6** on the C2-attached furan ring, previously found to be compatible with NanoLuc catalysis^23^, produced compound **10** that exhibited similar brightness (Extended Data Fig. 3b) but reduced solubility (Extended Data Fig. 3c). Combining these two strategies yielded compounds **11**–**13**. One of these compounds, **13**, was brighter than **6** in the brain by a factor of 1.5–2.0 (Extended Data Fig. 4). Finally, we tested **6** and **13** for toxicity in mice. A single i.p. injection of 4.2 µmol of **6** solubilized with 20 mg of P-407 was associated with minimal effects on heart and kidney tissues without weight loss (Supplementary Table 1), a more minor effect than observed after treatment with the same amount of FFz^16^. By contrast, one-time injection of 4.2 µmol of **13** caused severe kidney pathology and weight loss (Extended Data Fig. 5). Importantly, no signs of lung or brain damage were noted in mice receiving either substrate (Supplementary Table 1). Given its lower toxicity and its specific utility in the brain, we selected **6** for further use and named it CFz.

### Optimizing CFz dosing

**Extended Data Fig. 6:**
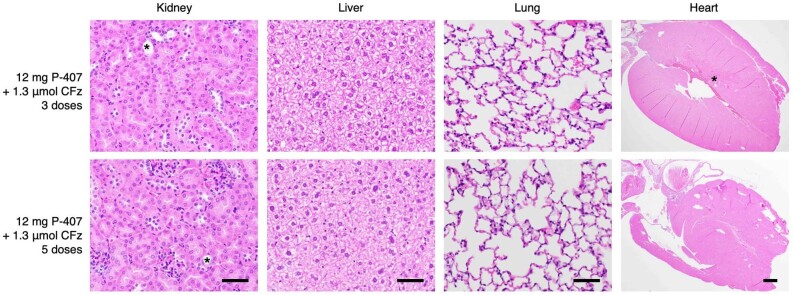
Minimal toxicity of repeated low-dose administration of CFz. Renal lesions were minimal to absent in mice receiving 3 or 5 daily i.p. injections of CFz (1.3 µmol). For kidney: asterisks, renal tubular dilation. For heart: asterisk, myocardial necrosis, and hemorrhage. No lesions were noted in the lungs across any groups. The selected images were representative dissection images with the most severe damage of three biological replicates for illustration purposes. Scale bars, 50 μm in kidney, liver, and lung, and 500 μm in heart. Detailed scores of histology analysis of all biological replicates were summarized in Supplementary Table 1.

We tested CFz formulations for compatibility with longitudinal imaging and for maximal brain brightness. After daily administration of 1.3 µmol of CFz (with 12 mg of P-407 excipient) for 3 or 5 d, effects on organ tissues were mild, and weight loss was limited to <5% (Extended Data Fig. 6 and Supplementary Table 1). Clear vesicles appeared in macrophages of the liver and spleen after 5 d (Supplementary Table 1). As similar effects are seen after PEG administration^25^, these vesicles may contain accumulated P-407 polymer. Taken together, these results suggest that CFz can be used for longitudinal imaging at a dose of 1.3 µmol with 12 mg of P-407.

**Extended Data Fig. 7:**
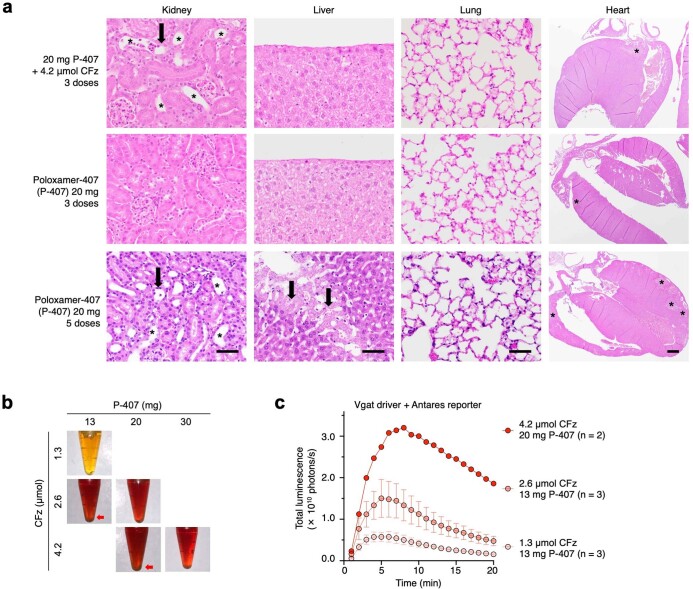
Limits of CFz dosing. **a**, Histology of organs after three daily i.p. injections of 4.2 µmol CFz with 20 mg P-407 or P-407 alone, or five injections of P-407 alone. For kidney: asterisks, renal tubular dilation; arrows, renal tubular degeneration and luminal sloughing. For liver: arrows, hepatic necrosis. For heart: asterisks, myocardial necrosis and hemorrhage. No lesions were noted in the lungs across any groups. Scale bars, 50 μm in kidney, liver, and lung, and 500 μm in heart. **b**, Representative photos of CFz injectable solution at various concentrations and P-407 vehicle ratios. Red arrows indicate the small fraction of precipitates after being separated by centrifuge. The experiment was repeated independently two times with similar results. **c**, Dose-dependent bioluminescence for CFz in mice doubly hemizygous for *Vgat*-IRES-*cre* and CAG-LSL-Antares.

As mentioned above, a single injection of 4.2 µmol of CFz with 20 mg of P-407 produced minimal toxicity. However, three daily i.p. injections of 4.2 µmol of CFz with 20 mg of P-407 were noticeably toxic to organs (Extended Data Fig. 7a and Supplementary Table 1). Thus, repeated imaging with this formulation would not be recommended. To understand the source of toxicity, we tested 20 mg of P-407 vehicle alone for 3 or 5 d and found mild effects on organs at 3 d but severe effects at 5 d (Extended Data Fig. 7a and Supplementary Table 1). Toxicity after three daily doses of 4.2 µmol of CFz and 20 mg of P-407 could thus arise from substrate alone or from the combination of substrate and excipient. In addition, three doses of 20 mg of P-407 alone induced the appearance of clear vesicles in macrophage cells (Supplementary Table 1), indicating that CFz is also not required for this response. In our hands, the amount of P-407 could not be reduced below 20 mg when dissolving 4.2 µmol of CFz, as a small amount of CFz residue already remained with 20 mg of P-407 but not 30 mg (Extended Data Fig. 7b).

Nevertheless, 4.2 µmol of CFz with 20 mg of P-407 produced 6.5-fold brighter bioluminescence from the brain than a 1.3-μmol dose (Fig. 2e and Extended Data Fig. 7c). This dose may be useful if maximizing sensitivity at a single time point is desired.

### Comparing the performance of luciferase systems in the brain

**Fig. 3:**
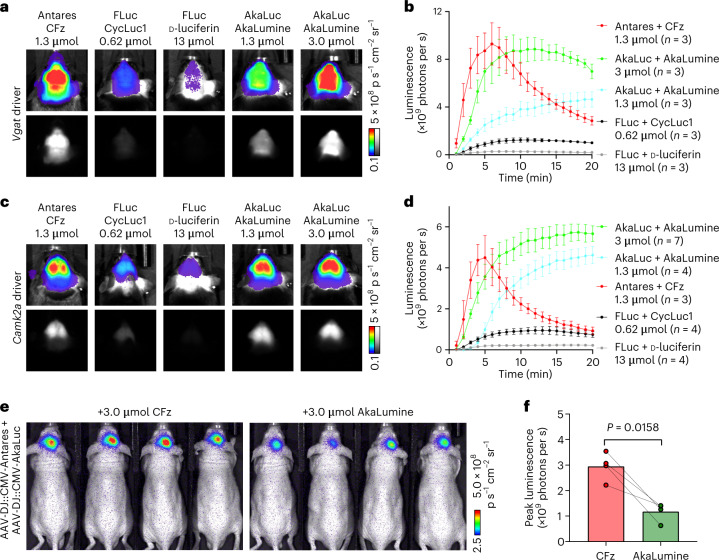
Comparison of CFz–Antares to other bioluminescent reporter systems. **a**–**d**, Comparison between Antares–CFz and other luciferase reporters in transgenic mice. **a**, Representative images of peak bioluminescence of reporters expressed in VGAT^+^ neurons. **b**, Bioluminescence intensity over time. Data are presented as mean values ± s.e.m.; *n* = 3 independent animals in each condition. **c**, Representative images of peak bioluminescence of reporters expressed in CaMKIIα^+^ neurons. **d**, Bioluminescence intensity over time. Data are presented as mean values ± s.e.m.; *n* = 7, 4, 3, 4 and 4 animals for AkaLuc with 3 µmol of AkaLumine, AkaLuc with 1.3 µmol of AkaLumine, Antares with 1.3 µmol of CFz, FLuc with 0.62 µmol of CycLuc or FLuc with 13 µmol of d-luciferin, respectively. **e**, Representative bioluminescence images of Antares–CFz and AkaLuc–AkaLumine in the hippocampus of J:NU mice co-infected with Antares- and AkaLuc-encoded AAV. **f**, Comparison of peak signal intensities. Data are presented as mean values ± s.e.m.; *n* = 4 animals, each of which was measured for both Antares–CFz and AkaLuc–AkaLumine signals. *P* values were determined by two-tailed Student’s paired *t*-test.

**Extended Data Fig. 8:**
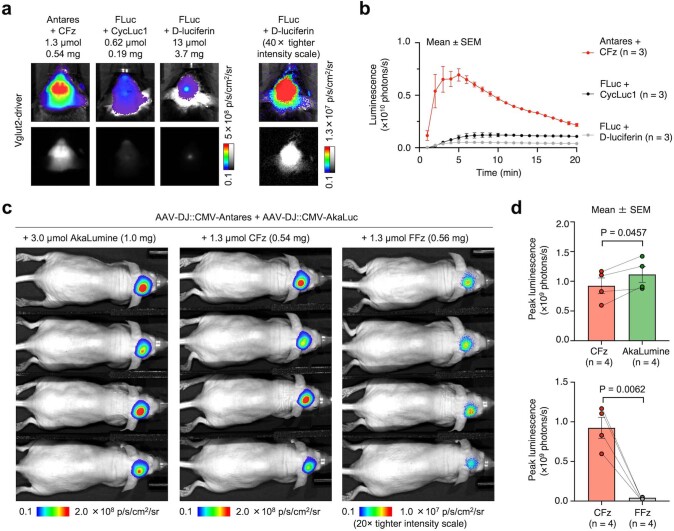
Additional comparisons between luciferase systems in the brain. **a-b**, Comparison between Antares-CFz and FLuc-D-luciferin/CycLuc1 in *Vglut2*-driven luciferase-expressing reporter mice. **a**, Representative images with peak bioluminescence from each substrate injection. **b**, Bioluminescence intensity in the brain over time. Data are presented as mean values ± SEM. n = 3 animals in each group. **c–e**, Comparison between Antares-CFz/FFz and AkaLuc-AkaLumine pairs in the hippocampus of J:NU mice co-infected with AAV expressing Antares and AAV expressing AkaLuc. **c**, Representative images with peak bioluminescence from each substrate injection. **d**, Mean peak signal intensities. Data are presented as mean values ± SEM. P = 0.0457 between Antares-CFz and AkaLuc-AkaLumine and P = 0.0062 between Antares-CFz and Antares-FFz by two-tailed Student’s paired t-test. n = 4 animals in each group.

We next compared brain signals from the Antares–CFz system to FLuc with either the standard d-luciferin substrate or the brighter CycLuc1 (ref. ^9^). Antares and FLuc Cre-dependent transgenes were recombined similarly into the *H11* locus and crossed to *Vgat-*IRES*-cre*, *Vglut2-*IRES*-cre* or *Camk2a-cre* drivers. Depending on the driver, Antares with 1.3 µmol of CFz produced 5- to 7.5-fold higher brightness than FLuc with CycLuc1, which in turn was 3- to 4-fold brighter than FLuc with d-luciferin (Fig. 3a–d and Extended Data Fig. 8a,b). Brain signal distributions appeared similar between these systems.

To compare Antares–CFz to AkaLuc–AkaLumine, we performed experiments with genetically matched expression and coexpression in the same animals. First, we created transgenic mice with CAG-LSL-AkaLuc recombined into the *H11* locus to obtain the same control elements and genomic context as the previously created CAG-LSL*-*Antares transgenic mice^16^ and crossed both to *Vgat-cre* or *Camk2a-cre* driver mice. In the resulting double hemizygotes, Antares with 1.3 µmol of CFz produced comparable peak signal and distribution patterns to AkaLuc with AkaLumine at the recommended 3.0-µmol dose (Fig. 3a–d). AkaLuc–AkaLumine produced a more sustained signal at either 3.0- or 1.3-µmol doses than Antares–CFz (Fig. 3b,d). Next, we expressed Antares and AkaLuc in the same brains by injecting equal titers of Antares- and AkaLuc-expressing adeno-associated virus (AAV) into the hippocampus. Three weeks later, these mice received CFz, FFz or AkaLumine on alternating days. CFz was brighter than FFz by at least an order of magnitude, and 1.3 µmol of CFz generated a comparable peak signal to 3.0 µmol of AkaLumine (Extended Data Fig. 8c,d). When CFz dose was increased to 3 µmol, brightness increased to about threefold higher than that of 3 µmol of AkaLumine (Fig. 3e,f). Thus, Antares–CFz brightness matches AkaLuc–AkaLumine at the 1.3-µmol dose suitable for daily imaging and exceeds it at a 3-µmol dose compatible with occasional imaging.

### Bioluminescent brain imaging in awake mice with CFz

**Fig. 4:**
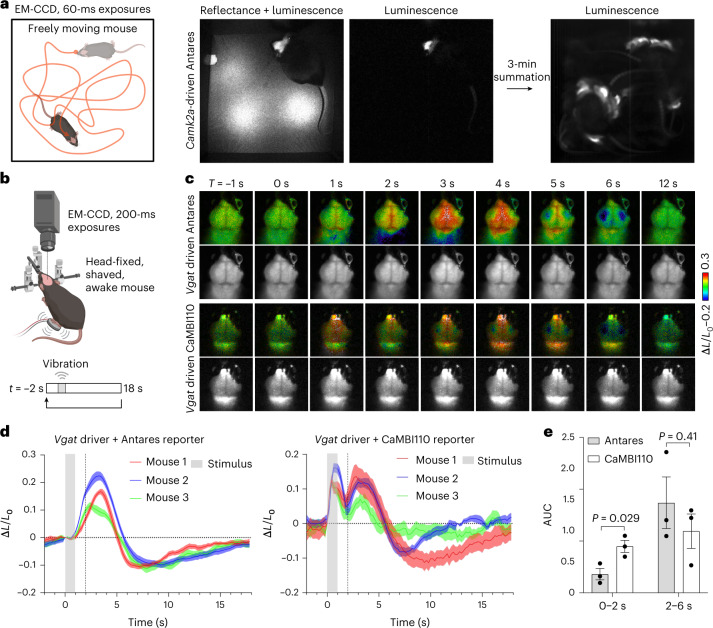
In vivo applications of CFz. **a**, Video-rate imaging of a mouse doubly hemizygous for *Camk2a*-*cre* and CAG-LSL-Antares transgenes. After i.p. injection of CFz, the freely moving mouse was monitored using an EM-CCD camera. Brightfield and luminescent images (60-ms exposure time for each) were alternately acquired, and a representative frame is shown. An integrated luminescent image spanning 3 min is shown. **b**, Scheme of bioluminescent brain imaging in head-fixed awake mice with foot vibrational stimulation. **c**–**e**, Comparison of stimulation-dependent brain bioluminescence responses between mice doubly hemizygous for *Vgat*-*cre* driver and CAG-LSL-Antares transgenes (*Vgat*-Antares) or CAG-LSL-CaMBI110 transgenes (*Vgat*-CaMBI110). **c**, Representative frames of an image sequence averaged and normalized from repeated 20-s periods of BLI in *Vgat*-Antares (top) or *Vgat*-CaMBI110 (bottom) transgenic mice. For each set of images, pseudocolored images (top) and grayscale images (bottom) are shown. **d**, Quantitation of timelapse, averaged and normalized cortical bioluminescence in **c**. Each line represents a mean value of *n* = 3 animals; the shaded area around each line represents s.e.m. The gray box indicates time of the vibrational stimulus applied to the mouse’s hind paw. Traces are averages of 20 stimulation cycles. **e**, Comparison of the area under the curve (AUC) values before and after the 2-s time point of each mouse in **d**. Data are presented as mean values ± s.e.m. *P* values were determined by two-tailed Student’s unpaired *t*-test.

Encouraged by the comparable performance of Antares–CFz to AkaLuc–AkaLumine, we attempted video-rate non-invasive imaging of Antares signal in the brains of freely moving mice. Video-rate tracking of AkaLuc signal in moving mouse brains had previously been demonstrated after intravenous (i.v.) AkaLumine administration^10^. We found that *Camk2a-cre*-driven Antares was clearly visible in freely moving mice with 60-ms exposure times after the easier i.p. administration of 1.3 µmol of CFz (Fig. 4a and Supplementary Video 1), enabling simultaneous visualization of mouse movement and brain bioluminescence.

**Extended Data Fig. 9:**
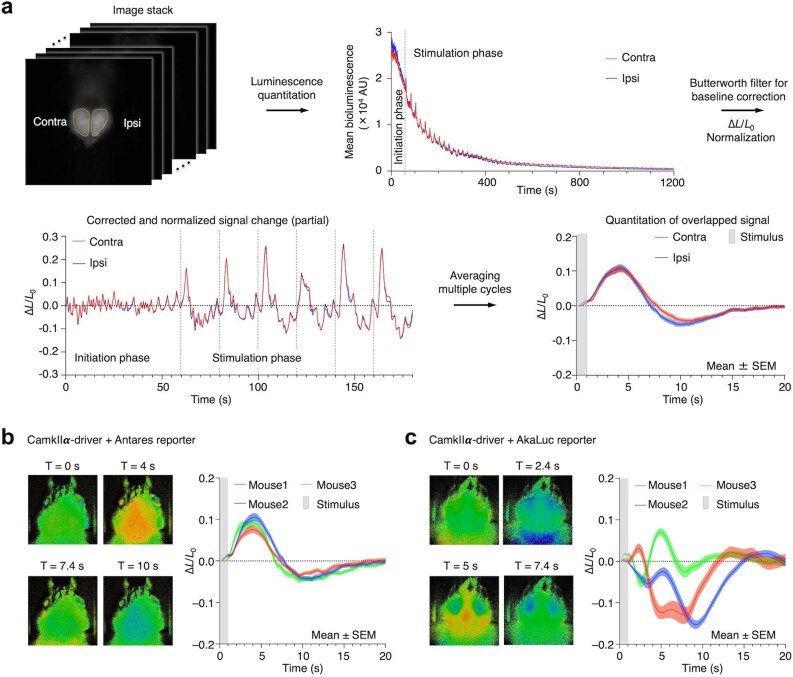
Effects of stimulation on bioluminescence from constitutive luciferases in awake mice. **a**. Example of data processing pipeline for the stimulation and imaging on head-fixed awake mice. **b**, Stimulation-dependent brain bioluminescence responses of mice doubly hemizygous for a *CaMKIIα-cre* driver and CAG-LSL-Antares reporter. **c**, Stimulation-dependent brain bioluminescence responses of mice doubly hemizygous for a *CaMKIIα-cre* driver and CAG-LSL-AkaLuc reporter. For **b** and **c**: Left, representative frames of an image sequence averaged and normalized from repeated 20-s periods of BLI; right, averaged and normalized cortical bioluminescence. Traces in **a–c** are averages of 57 stimulation cycles.

As bloodflow in the brain is induced by neuronal activity, and the substrates and oxygen required for the luciferase reaction are delivered via the blood, Antares–CFz and AkaLuc–AkaLumine signals could be inherently influenced by activity. To test this, we applied sensory stimulation in the form of a 1-s vibration to one foot while recording Antares or AkaLuc at 5 frames per s (Fig. 4b). Mice were head fixed to reduce movement or orientation artifacts, and CFz was retro-orbitally (r.o.) administered, as the ventral area was inaccessible after head fixation. In mice expressing Antares in the forebrain under *Camk2a-cre* control, stimulation was followed by bilateral increases in bioluminescence peaking 2 to 4 s later (Extended Data Fig. 9a,b). The bilateral pattern likely indicates widespread neuronal activation from an arousal response, as previously observed in functional magnetic resonance imaging (fMRI)^26,27^. A similar response was seen in mice expressing Antares under *Vgat-IRES-cre* control (Fig. 4c,d and Supplementary Video 2). The direction (increase) and duration (4 s) of these changes were consistent between mice, suggesting that they may be reliable indications of changes in bloodflow, similar to fMRI^28^.

Stimulus-induced signal changes also occurred in *Camk2a-cre-*driven AkaLuc mice (Extended Data Fig. 9c). However, their direction (both upward and downward) and duration (4 to 13 s) varied greatly between mice, making interpretation more difficult. One possibility is that AkaLumine initially distributes to tissue compartments, from where its release may then be influenced by body movements. Nevertheless, taken together, these results indicate that bioluminescence signals from both Antares–CFz and AkaLuc–AkaLumine systems are influenced by sensory stimuli, likely via hemodynamic effects.

### Non-invasive calcium imaging in the brain with CaMBI and CFz

As previous studies have revealed neuronal calcium influx preceding hemodynamic changes in the mouse cortex^29–31^, we next tested whether a NanoLuc-based calcium reporter can provide faster indications of brain activity non-invasively. Several NanoLuc-based calcium indicators have been developed, including GeNL(Ca^2+^)^15^, CalFluxVTN^32^, GLICO^33^, LUCI-GECO1 (ref. ^34^), CalBiT^20^, H-Luc labeled with the chemical dye MaPCa^35^ and Orange CaMBI^36^. Orange CaMBI110 consists of Antares with a calcium-sensing module inserted into the NanoLuc domain and exhibits an eightfold responsiveness to calcium and a dissociation constant of 110 nM. As we had already generated transgenic mice expressing Orange CaMBI110 in a Cre-dependent manner^16^, we used them to test whether CFz would enable non-invasive calcium imaging from genetically defined neurons in the brain.

We performed sensory stimulation and BLI on mice hemizygous for *Vgat-*IRES*-cre* and CAG-LSL*-*CaMBI110 transgenes^36^. Indeed, in addition to the previously observed hemodynamic response occurring 2–6 s after stimulation, we also observed an earlier bioluminescence peak 1 s after stimulation (Fig. 4c,d and Supplementary Video 3). Quantitation confirmed that the CaMBI110 and Antares mice showed similar late responses (2–6 s after stimulation), but only the CaMBI110 mice exhibited a significant early response (0–2 s; Fig. 4e). Thus, the early bioluminescent response revealed solely by CaMBI110 likely represents calcium influx, while the late response shared by CaMBI110 and Antares is presumably due to hemodynamic changes in substrate or oxygen supply. Taken together, these results indicate that CFz and CaMBI110 enable non-invasive detection of neuronal calcium activity.

**Extended Data Fig. 10:**
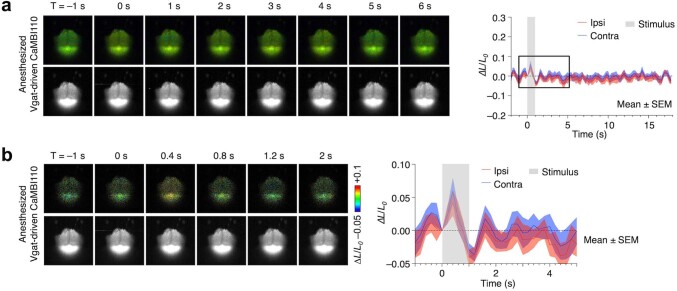
Orange CaMBI110 responses to vibrational stimulation in anesthesized mice. **a**, Left, Representative frames of an image sequence averaged and normalized from 20 cycles of BLI after vibrational stimulus in anesthetized mouse doubly hemizygous for *Vgat-cre* and CAG-LSL-CaMBI110 transgenes. Top, pseudo-colored images; bottom, grayscale images. Right, normalized mean cortical bioluminescence. Gray box, the vibrational stimulus applied to the mouse’s hind paw. Shaded area, error bars showing s.e.m. **b**, Left, expanded time series within a shorter time interval surrounding the stimulus. Right, normalized mean traces from the boxed region in **a**. Traces in **a–b** are averages of 20 stimulation cycles.

Unexpectedly, the early CaMBI110-specific response that we attributed to neuronal activity also exhibited a bilateral pattern, whereas the direct cortical response to the stimulus might be expected to be stronger in the hemisphere contralateral to the stimulus. One explanation is that most of the neuronal activation is from second-order activation of a pancortical arousal response, dominating over any unilateral primary response. To test this, we performed stimulation and BLI on the same reporter mice in an anesthetized state (Extended Data Fig. 10). The amplitudes of bioluminescence responses were substantially decreased compared to those observed in the awake state, consistent with the hypothesis that the bioluminescence response in awake mice mainly reflected an arousal response.

## Discussion

In this study, we developed a new substrate, CFz, that enhances the sensitivity of BLI in the brain with NanoLuc-based reporters by at least 2.5-fold compared to the previously best substrate at standard doses, while a further 6.5-fold improvement in brightness could be achieved with occasional higher dosing. The combination of Antares luciferase with CFz achieved comparable brightness in the brain to AkaLuc–AkaLumine, previously the brightest BLI system for brain imaging. With i.p. injected CFz, we demonstrate the ability to visualize Antares-expressing cells in the brain non-invasively at a video rate. We also use CFz with the calcium-modulated bioluminescent indicator CaMBI110 to perform genetically specified non-invasive imaging of sensory-evoked neuronal activity in the mouse brain.

Importantly, because CFz is an excellent substrate for NanoLuc, it can be immediately applied in a plug-and-play manner to existing NanoLuc-based reporters, such as reporter-encoded AAV vectors, reporter cell lines and transgenic animals. That is, these reporters can now be used in the brain with several-fold higher sensitivity than possible with previous NanoLuc substrates.

To facilitate rapid evaluation and characterization of luciferase substrates in mouse brains, we used transgenic mice as the screening platform in this study. Transgenic reporter mouse lines provide major advantages for substrate screening. First, once the transgenic mouse lines are successfully established, they become a stable supply without the need for additional surgery or other cost- and time-consuming procedures. Second, stable expression of reporters can reduce the sources of variability, such as day-to-day variation in reporter expression level or technical variation introduced during procedures, thus increasing the statistical relevance and reducing the number of mice needed. Most importantly, transgenic mice have an intact BBB, while other models with surgery involved might disrupt the BBB integrity, which could affect the accuracy of the evaluation.

We have focused on substrates that can be delivered by i.p. injection, which is easier to perform compared to i.v. or r.o. injections and produces brighter bioluminescence than subcutaneous injection. Typically, i.v. injection results in higher peak bioluminescence but also faster decay^37^. In the head-fixed mouse brain imaging experiment, due to the inaccessibility of the mouse abdomen after head fixation, r.o. was adopted as the route of administration, and signal features similar to that of i.v. injection were observed (Extended Data Fig. 9a). It would be of interest to directly and systemically compare the in-brain performance of CFz between these different routes of administration.

It is useful to remember that substrates behave like drugs in living animals^38^. Beyond the in vitro characterizations, substrates and formulations should be comprehensively evaluated for pharmacodynamics and toxicity. Here, both substrate candidates CFz and compound **13** were examined for in vivo toxicity, and CFz was chosen on the basis of less toxicity after a single 4.2-µmol dose (Supplementary Table 1). For serial BLI, daily i.p. injection of 1.3 µmol of CFz for up to 5 d appears well tolerated. By contrast, daily i.v. injection of 60 µg (0.16 µmol) of Fz for 7 d caused liver and lung damage^39^, while 20 µg i.v. per day showed minimal toxicity. Five daily doses of 20 mg of P-407 excipient alone caused notable toxicity, so the future identification of other excipients could enable repeated BLI with high-dose CFz. Regarding other luciferins, while AkaLumine was developed and demonstrated superior in vivo performance^10,40^, follow-up studies revealed noticeable toxicity of the Akalumine solution to the skin and heart, probably due to the acidity of the administered solution^41,42^. Finally, few systematic studies on the in vivo toxicity of d-luciferin have been reported, and it has been speculated that high-concentration usage may be toxic as well^43,44^.

The kinetics of light production in the brain should also be considered when choosing a luciferase–substrate system. Antares–CFz brain signals rise and decay more quickly than AkaLuc–AkaLumine signals. The slower AkaLuc–AkaLumine signals are likely due to slower AkaLumine permeation into the brain, as AkaLuc–AkaLumine signals in the body were faster^16^. Slow signal decay could be advantageous for timelapse imaging. However, for the particular task of tracking hemodynamic responses to brain stimulation, AkaLuc–AkaLumine responses to stimulation were highly variable (Extended Data Fig. 9b,c), suggesting that permeation of AkaLumine in the brain may be too slow in some mice to report hemodynamic responses occurring on the timescale of seconds. By contrast, Antares–CFz reliably reported stimulation-induced responses. In addition, the faster kinetics of CFz are advantageous for multiplex BLI, as the fast decay of CFz allows a second BLI system, such as AkaLuc–AkaLumine, to be used without a long delay.

In our study, the brightest substrate outside the brain, FFz, was not the brightest substrate in brain neurons. In addition, biochemical measurements of CFz and Fz PK measurements showed similar concentrations in brain tissue, although CFz was several-fold brighter. Thus, neither performance outside the brain nor PK measurements in the brain could predict the better performance of CFz. These results demonstrate the importance of empirically testing luciferase substrates for brain brightness and suggest that other chemical compounds such as drugs could also demonstrate a lack of correlation between measurable concentrations in brain tissue and activity at their target enzymes (pharmacodynamics).

In this study, Antares and AkaLuc signals revealed a correlation between sensory stimulation and changes in bioluminescence intensity in head-fixed awake mice (Fig. 4b–d and Extended Data Fig. 9b,c). We hypothesize that hemodynamic changes may underlie these responses because luciferases rely on blood circulation to deliver the required substrate and oxygen, and the flow of oxygenated blood is induced by neuronal activities in the brain^28^. This hypothesis is supported by the observation that the correlation is neither driver specific nor reporter specific. In particular, cortical AkaLuc BLI also revealed a stimulus-dependent signal fluctuation (Extended Data Fig. 9c), although AkaLuc belongs to a different category of luciferases from Antares and is ATP dependent^1,2^. Thus, researchers should be cautious about the possible interference from hemodynamic responses when performing timelapse BLI with frequent (<10-s) intervals. However, BLI of constitutive luciferases can be developed into an alternative method to study hemodynamics in vivo, producing similar information as fMRI but without the need for expensive machinery. However, BLI modulation by hemodynamics might be brain region dependent because it was not observed in the basolateral amygdala with a foot-shock stimulus^45^.

Finally, our results with the calcium-modulated bioluminescent indicator Orange CaMBI110 revealed a positive sensory-induced response preceding the hemodynamic response (Fig. 4c–e and Supplementary Video 3). This is consistent with the established relationship between neuronal activation and a subsequent hemodynamic response^29–31^. In addition, the similarity in the amplitude of the presumed calcium and hemodynamic signals (~10%) suggests that any further CaMBI improvements could generate calcium-specific signals above hemodynamic responses. An improved CaMBI would also improve the chance of detecting highly localized neuronal activities. A recent study on lightly anesthetized Thy1-GCaMP6f mice has revealed that local neuronal responses evoked by vibrational stimuli are low in amplitude (contralateral: 1.6% ∆*F*/*F*; ipsilateral: 0.8% ∆*F*/*F*)^46^. Thus, improving responses of CaMBI-type reporters to match or surpass fluorescent calcium indicators is an important direction for bioluminescent activity imaging.

In summary, the development of CFz should enable sensitive NanoLuc-based bioluminescence imaging in the brains of preclinical animal models. The desirable bioluminescence features of Antares with CFz and AkaLuc with AkaLumine and the high orthogonality between these two bioluminescent reporter systems suggest several attractive avenues for future applications of dual imaging in the brain. For example, two-population brain imaging can be applied to study the interaction between immune cells and cancer cells or between viruses and immune cells. Another possibility would be to use these orthogonal luciferases as transcriptional reporters of different biochemical pathways in the same cell or as reporters of the same pathway in different cell types in the brain.

## Methods

### Compound characterization

All new compounds were prepared using the synthetic route described in scheme 1 (Supplementary Note)^16,47^. Fz, HFz and FFz have been previously reported^12,16^. Characterization (^1^H-NMR, ^13^C-NMR and MS) of the newly synthesized compounds can be found in the Supplementary Information.

### Solid formulation for use in whole-animal imaging

Stock samples of solid formulated Fz analogs were prepared using the following protocol. First, P-407 (Pluronic F127) was placed into a screw-cap glass vial. The polymer was then heated to 75 °C in a water bath until melted. Fz analogs dissolved in ethanol were added to the hot polymer, mixing well with a thin spatula. Additional rinses of Fz analogs in ethanol were added to the hot polymer for quantitative transfer. The solvent was then removed under vacuum. This concentrated sample was diluted with water to make a dispensing mixture of 4–9 mM Fz analogs in water. Next, this aqueous stock was aliquoted into glass vials, frozen and lyophilized overnight to give a yellow cake. For i.p. injection into mice, DPBS (Corning, 21-031-CV) was added to the vial and rocked to create a clear solution.

### Bioluminescence imaging of transgenic mice for substrate comparison

Animal procedures were performed in compliance with United States Department of Agriculture and NIH ethical regulations and were approved by the Stanford Institutional Animal Care and Use Committee. Mice were housed at 45–65% humidity and 20–24 °C on a 12-h light/12-h dark cycle.

Transgenic reporter mice with LSL-CAG-luciferase (Antares, FLuc or AkaLuc) inserted at the *H11* locus (*H11P*) were generated by φC31-mediated recombination in blastocysts^48^ by the Stanford Transgenic, Knockout and Tumor Model Center. For substrate comparisons, 2- to 4-month-old transgenic mice doubly hemizygous for a *Camk2a-cre* (Jackson Laboratory, 005359), *Vgat-*IRES*-cre* (Jackson Laboratory), *Vglut2-*IRES*-cre* (Jackson Laboratory, 016963) or *Alb-cre* (Jackson Laboratory, 003574) driver and a CAG-LSL*-*Antares, CAG-LSL*-*AkaLuc or CAG-LSL*-*FLuc reporter were anesthetized by inhalation of isoflurane for 5 min. An electric shaver was used to remove scalp hair in *Camk2a-cre*, *Vglut2-*IRES*-cre* or *Vgat-*IRES*-cre* mice or abdominal hair in *Alb-cre* mice. Nair depilatory cream (Church and Dwight) was then applied to the shaved area for 1 min, and remaining hair was removed with a piece of sterile gauze. The area was then rinsed with DPBS and cleaned with 70% ethanol with a piece of sterile gauze.

For mice expressing Antares, indicated amounts of Fz were dissolved in 480 µl of a PEG-300-based formulation consisting of 10% glycerol, 10% ethanol, 10% hydroxypropylcyclodextrin and 35% PEG-300 in water, or indicated amounts of lyophilized Fz analogs with P-407 vehicle were reconstituted in 150–500 µl of DPBS buffer. For mice expressing FLuc, indicated amounts of d-luciferin were dissolved in PBS. For mice expressing AkaLuc, 3 μmol (1.0 mg) of AkaLumine-HCl (Sigma-Aldrich) was dissolved in 150 μl of 0.9% NaCl. Substrates were then injected into the peritoneal cavity in the left lower quadrant, with the maximum injectable volume at 480 µl. Immediately after substrate injection, bioluminescent images were acquired in an Ami HT bioluminescence imaging system running Aura software version 3.1 or 4.0 (Spectral Instruments Imaging) for 15–30 min at intervals of 1 min. For comparing substrates at 1.3 µmol, the following imager settings were used: emission filter open, field of view of 25 cm, f-stop of 1.2, binning of 2 × 2 and exposure time of 2 s. For comparison with >1.3-µmol substrates, the following settings were used: emission filter open, field of view of 25 cm, f-stop of 1.2, binning of 1 × 1 and exposure time of 1 s. Pseudocolor images and intensity scale bars were generated with Aura software. Integrated luminescence was quantified using the Fiji version of the ImageJ program (NIH)^49^, with further statistical analysis performed in GraphPad Prism (version 9.0.0).

### Enzymatic analysis of Antares and Fz analogs

Two milliliters of LB broth with 100 µg ml^–1^ ampicillin was inoculated with a single colony of clone ATG-3802 (Antares codon optimized for *Escherichia coli*) and grown overnight at 37 °C. On the following day, the overnight culture was diluted 1:100 into a flask containing 50 ml of Terrific Broth and 100 µg ml^–1^ ampicillin. The sample was grown at 37 °C with shaking (275 r.p.m.) for 4 h. The culture was chilled on ice, and 1% rhamnose was added to induce protein expression. The culture was placed in a 15 °C shaker (275 r.p.m.) and grown for 48 h. Cells were collected by centrifugation and resuspended in 10 ml of 100 mM HEPES (pH 7.5), 150 mM NaCl, 10 mM imidazole and 1 mg ml^–1^ lysozyme. The resuspended cells were sonicated on ice (5 s on, 5 s off for 2 min, 12 amplitude). The sample was centrifuged (12,000*g* for 15 min), and 1 ml of HisLink resin (Promega) was added to the cleared lysate. Samples were incubated at 4 °C on an orbital mixer for 2 h. After incubation, the resin was washed three times with HisLink wash buffer (Promega). After the final wash, the sample was eluted (incubated 3 min with mixing) two times with 1 ml of HisLink elution buffer (Promega). The sample was dialyzed (10-kDa molecular weight cutoff, Thermo Fisher Slide-A-Lyzer) for 24 h in TBS and 50% glycerol.

To measure emission spectra with Fz and new substrates, 50 μl of a solution of 100 pM Antares protein and 0.01% (wt/vol) bovine serum albumin (BSA; Promega) in DPBS (Life Technologies, 14190) was mixed with 50 μl of a solution of 30 μM substrate and 0.01% BSA in DPBS in triplicate. Emission from 400 to 750 nm was then measured using an Infinite M-1000 luminometer (TECAN).

To quantify luminescence as a function of substrate concentration, substrates were diluted to 30 μM and serially diluted in twofold steps into DPBS with 0.01% BSA in triplicate. Fifty microliters of each substrate dilution was then mixed with 50 μl of a solution of 20 pM Antares protein in DPBS with 0.01% BSA. Luminescence was measured immediately using a GloMax-Multi+ luminometer (Promega). *K*_m_ and *V*_max_ were then calculated using the Michaelis–Menten non-linear regression function in the program Prism (GraphPad).

### In vivo substrate pharmacokinetics

PK measurements were performed at Shanghai ChemPartner for Fz and compound **6** in 6- to 8-week-old overnight-fasted male C57BL/6 mice. Fz and compound **6** were formulated as clear solutions in 10% ethanol, 10% glycerol, 10% hydroxypropyl-β-cyclodextrin (Sigma-Aldrich), 35% PEG-300 and 35% water. Freshly prepared injectable doses (i.p., 20 mg per kg body weight for Fz and 21.6 mg per kg body weight for compound **6**) were administered at 20 ml kg^–1^. Each of the two measurement series consisted of four mice and two time points (series 1, predose and 0.083 h; series 2, 0.083 h and 0.167 h).

At each time point, ~90 µl of blood was collected into a K_2_EDTA tube via facial vein bleeding. Plasma samples were put on ice and centrifuged to obtain plasma samples (2,000*g* for 5 min at 4 °C) within 15 min of collection. The plasma samples were stored at –70 °C until analysis. For analysis, 20 µl of plasma sample was added to 20 µl of stabilizer (80:20 (vol:vol) mixture of 200 mg ml^–1^ EDTA and 100 mM l-ascorbic acid in water).

At the terminal point of each series, a midline incision was made in the animal’s scalp, and the skin was retracted. The skull overlying the brain was removed. The whole brain was collected, rinsed with cold saline, dried on filtrate paper, weighed and snap-frozen by placing it into dry ice. No abnormal symptoms were observed during the studies. The brain samples were homogenized with 4 ml of stabilizer (80:20 (vol:vol) mixture of 200 mg ml^–1^ EDTA and 100 mM l-ascorbic acid in water) per gram of tissue. The above samples (plasma or brain) were mixed with 120 µl of internal standard (Glipizide, 100 ng ml^–1^) in acetonitrile. The mixture was shaken for 10 min and centrifuged at 5,200*g* for 10 min at 4 °C. Twenty microliters of supernatant was transferred to a new plate, and then 20 µl of water was added. Compound concentrations were quantified using LC–MS/MS detection.

Bioanalysis was performed on a Shimadzu UPLC LC-30AD system coupled with an AB SCIEX Triple Quad 6500^+^ mass spectrometer. Mass parameters were performed in positive mode with the ESI source ionization under MRM mode. The separation of the test article was achieved using a Waters Acquity UPLC BEH C18 column (2.1 × 50 mm, 1.7 µm) with a flow rate of 0.60 ml min^–1^ at 50 °C. Mobile phase A was 0.025% formic acid and 1 mM ammonium acetate in water, and phase B was 0.025% formic acid and 1 mM ammonium acetate in methanol. The injection volume was 0.5 µl.

PK parameters were determined by the non-compartmental model of the non-compartmental analysis tool, Pharsight Phoenix WinNonlin 8.2 software. To calculate *T*_half_, time points were automatically selected by the ‘best fit’ model for terminal half-life estimation as the first option. Manual selection was applied when ‘best fit’ could not well define the terminal phase. When the number of detected time points after *T*_max_ was less than 3, the terminal half-life and the area under the plasma concentration-time curve from zero to infinity were not calculated.

### Necropsy and histopathology

Male C57BL/6 mice of 2 months of age were killed by CO_2_ asphyxiation and cardiac exsanguination. All mice were weighed, and a complete gross examination of the external and internal organs was performed. The following tissues were collected and immersion fixed in 10% neutral-buffered formalin for 72 h: liver, kidneys and lung. Formalin-fixed tissues were processed routinely, embedded in paraffin, sectioned at 5 μm and stained with hematoxylin and eosin. All slides were blindly evaluated for histologic treatment-related effects (Supplementary Table 1) by a board-certified veterinary pathologist.

### Comparison of bioluminescence in luciferase-transduced mouse brain

For AAV packaging, pAAV-CMV-AkaLuc-P2A-mNeonGreen-WPRE plasmid and pAAV-CMV-Antares-P2A-mNeonGreen-WPRE were amplified and purified by a PureLink Expi endotoxin-free maxi plasmid purification kit (Invitrogen) and were sent to Stanford Gene Vector and Virus Core to produce and titer AAV.DJ vectors. Under sterile conditions, 12-week-old male J:NU mice (Jackson Laboratory, 007850) were anesthetized with isoflurane and secured in a stereotaxic frame (RWD Life Science), and a hole the size of the needle was drilled through the skull. A Hamilton syringe with a 33-gauge needle was inserted at anteroposterior −2.2 mm, mediolateral +1.7 mm and dorsoventral −1.7 mm. After a 2-min wait, the needle was pulled back 0.3 mm, and 1 µl containing 1.4 × 10^10^ genome copies each of AkaLuc- and Antares-expressing AAV was injected at a speed of 0.1 μl min^–1^ using a syringe pump (KD Scientific). The needle was left in place for 3 min after each injection to minimize upward flow of viral solution. Two to 3 weeks after AAV infection, mice were injected i.p. with the indicated amount of CFz, Akalumine or FFz and imaged on the Ami HT (binning of 1 × 1 and exposure time of 10 s).

### Video-rate mouse brain bioluminescence imaging

A transgenic mouse doubly hemizygous for *Camk2a-cre* and LSL-CAG-Antares (male, 16 weeks old) with scalp hair shaved and naired was imaged after i.p. administration of 4.2 µmol of CFz with 20 mg of P-407 in 480 µl of DPBS. Bioluminescence imaging was performed using a homemade imaging black box equipped with an iXon ultra 888 electron-multiplying CCD (EM-CCD) camera (Andor) and a 50-mm focal length, 67-mm diameter and f/0.95 aperture lens (Speedmaster). During imaging, the mouse was allowed to move freely in an enclosed area (14.5 × 14.5 cm). A bioluminescent image and a brightfield image were alternately acquired with the following camera settings in the Andor Solis software: exposure time of 60 ms, binning of 2 × 2 and vertical clock voltage amplitude of 4.

### Bioluminescence imaging of hemodynamics and calcium dynamics in awake mouse brain

Transgenic mice doubly hemizygous for *Camk2a-cre* or *Vgat-*IRES*-cre* and LSL-CAG*-*Antares or LSL-CAG*-*CaMBI110 or LSL-CAG-AkaLuc (mixed male and female, 7–12 weeks old) with scalp hair shaved and naired were fixed on a SG-4N head holder (Narishige International), followed by r.o. injection of 0.33 (for Antares) or 1.3 µmol (for CaMBI110) of CFz with 12:1 (wt/wt) P-407 in 180 µl of DPBS or the recommended amount of AkaLumine in saline. One minute afterward, bioluminescence imaging was performed using a homemade imaging black box equipped with an iXon Ultra 888 EM-CCD camera (Andor) and a lens (Speedmaster 50-mm f/0.95, 67-mm diameter, Zhongyi). Image acquisition was performed with Andor Solis software using the following camera settings: exposure time of 200 ms, frame rate of 5 Hz and binning of 4 × 4. One minute after the start of image acquisition, 57 mechanical foot vibrational stimuli cycles (1 s on and 19 s off) were applied to the left hindlimb using a DZS electrical mini vibration motor gated by an AFG 31000 arbitrary function generator (Tektronix). The function generator and the EM-CCD were synchronized using an NI USB-6221 multifunction I/O device (National Instruments) controlled by MATLAB.

Image sequences were analyzed using MATLAB. Specifically, each frame of the image sequence was first subtracted for dark baseline, and a binary mask highlighting the mouse head with strong bioluminescence intensity was generated based on the intensity of each pixel. The values of the pixels inside the binary mask remained unchanged, while the values of pixels outside were set to be NaN. To account for the decaying nature of the bioluminescence intensity after substrate injection, the mean pixel intensity inside the binary mask was calculated for each frame of the image sequence, and the obtained mean intensity dynamics were smoothed using a Butterworth lowpass filter. Each frame of the image sequence was then divided by the corresponding value of the smoothed mean intensity dynamics for baseline correction. Afterward, the image sequence was split into trials starting 2 s before a foot vibration stimulus and spanning 20 s (the cycle length). Pixel values in the image sequence for each trial were then normalized to the average values within the first second of the trial, followed by averaging of either all 57 trials (in the early experiments of Extended Data Fig. 9) or the first 20 trials (in all other experiments). For image pseudocoloring, a lookup table was applied in Fiji to represent ∆*L*/*L*_0_ values of –0.3 and 0.3, and intensity of the pseudocolored image was modulated by the normalized luminescence intensity of each frame.

### Statistics

Based on standard deviation values measured in preliminary experiments, a power calculation using G*Power software revealed an 80% likelihood of detecting a 50% difference in brain signals, measured relative to the lower value, with three animals. We thus aimed for three animals per condition, but if more animals were available from breeding, they were included as well. Normality of data was assessed by Shapiro–Wilk test in Prism version 9.0.0 (GraphPad). Student’s *t*-tests and one-way analysis of variance (ANOVA) with Tukey’s post hoc tests were also performed in Prism.

### Reporting summary

Further information on research design is available in the Nature Portfolio Reporting Summary linked to this article.

## Online content

Any methods, additional references, Nature Portfolio reporting summaries, source data, extended data, supplementary information, acknowledgements, peer review information; details of author contributions and competing interests; and statements of data and code availability are available at 10.1038/s41589-023-01265-x.

## Supplementary information

Supplementary Information Supplementary Tables 1–3.

Reporting Summary

Supplementary Video 1 Bioluminescence imaging of Antares luciferase in CaMKIIα-positive neurons in a freely moving mouse. After i.p. injection of CFz, a mouse expressing Antares under the control of a *Camk2a-cre* driver was monitored during free movement using an EM-CCD camera. The head was shaved, but no surgery was performed; bioluminescence was acquired through intact skull and skin. Brightfield and luminescent images (60-ms exposure time for each) were alternately acquired. The video was recorded at a resolution of 512 × 512 pixels at 8.33 frames per s (fps) for 3 min. A 1-min segment played back at 17 fps (~2× actual speed) is shown; left, brightfield; right, luminescence. Related to Fig. 4a.

Supplementary Video 2 Non-invasive bioluminescence brain imaging of Antares luciferase in VGAT^+^ neurons. After r.o. injection of CFz, a mouse expressing Antares under the control of a *Vgat-cre* driver was imaged during head fixation. The head was shaved, but no surgery was performed; bioluminescence was acquired through intact skull and skin. Images were acquired at 5 fps of 20 repeats of 1-s vibrational stimulation to one foot followed by 19 s of rest (Fig. 4b), and the 20 repeats were averaged; left, raw bioluminescence image in grayscale; right, pseudocolor. Related to Fig. 4c–e.

Supplementary Video 3 Non-invasive bioluminescence brain imaging of CaMBI110 in VGAT^+^ neurons. After r.o. injection of CFz, a mouse expressing CaMBI110 under the control of a *Vgat-cre* driver was imaged during head fixation. The head was shaved, but no surgery was performed; bioluminescence was acquired through intact skull and skin. Images were acquired at 5 fps of 20 repeats of 1-s vibrational stimulation to one foot followed by 19 s of rest (Fig. 4b), and the 20 repeats were averaged; left, raw bioluminescence image in grayscale; right, pseudocolor. Related to Fig. 4c–e.

## Data Availability

The main data supporting the findings of this study are available within the article and its Supplementary Information. The raw data generated in this study are available from the corresponding author upon request.

## Code availability

The MATLAB code used for image processing in this work is available from the corresponding author upon request.
