# Supplementary material for: An optimized bioluminescent substrate for non-invasive imaging in the brain
Source: Nat Chem Biol. 2023 Feb 9;19(6):731–9. doi: 10.1038/s41589-023-01265-x (PMC10229426; doi:10.1038/s41589-023-01265-x)

nature chemical biology

Article

https://doi.org/10.1038/s41589-023-01265-x

An optimized bioluminescent substrate for non-invasive imaging in the brain

In the format provided by the authors and unedited

	1 day<br>4.2 μmol **13**<br><br>20 mg P-407	1 day<br>4.2 μmol **6**<br>(CFz)<br>20 mg P-407	3 days<br>1.3 μmol **6**<br>(CFz)<br>12 mg P-407	5 days<br>1.3 μmol **6**<br>(CFz)<br>12 mg P-407	3 days<br>4.2 μmol **6**<br>(CFz)<br>20 mg P-407	3 days<br>20 mg P-407	5 days<br>20 mg P-407
Liver							
LC	0 0 0	0 0 0	0 0 0	0 0 0	0 0 0	0 1 0	0 0 0
HN	0 0 0	0 0 0	0 0 2	0 0 0	0 0 0	0 0 0	2 4 2
Kidney							
RTDR	3 2 3	0 0 1	1 1 1	1 1 0	3 2 2	0 0 0	1 1 1
RTD	2 2 3	0 1 1	1 0 1	0 0 0	3 2 3	0 0 0	3 3 1
Heart							
HMNH	0 0 0	1 0 0	1 1 1	0 0 1	2 1 2	1 0 2	4 4 3
HT	0 0 0	1 0 0	0 0 0	0 0 1	1 1 1	1 0 1	1 1 1
HPMC	0 0 0	0 0 0	0 0 0	0 0 1	0 0 1	1 0 1	1 1 1
Brain							
BMH	0 0 0	0 0 0	0 0 0	0 0 0	0 0 0	0 0 0	1 1 0
Total organ score	5 4 6	2 1 2	3 2 5	1 1 3	9 6 9	3 1 4	13 15 9
RES							
KCHMV	0 0 0	0 0 0	0 0 0	2 2 2	2 2 2	2 2 1	4 4 4
SRPHMV	0 0 0	0 0 0	0 0 0	1 1 1	1 1 1	1 1 1	1 1 1
Δ weight (%)	–7.9 –7.2 –6.7	–0.7 +0.3 +1.4	–2.2 –0.4 –1.1	–3.2 –4.6 –1.8	–8.3 –3.7 –3.0	–3.1 –0.7 –5.8	–19 –20 –18

Supplementary Table 1 | Individual Histologic Scores Across Treatment Groups and Histologic Scoring Criteria. LC, Liver capsule: 0, normal; 1, segmental subcapsular necrosis +/- neutrophilic infiltrate. HN, hepatocellular necrosis: 0, absent; 1, minimal; 2, mild; 3, moderate; 4, severe. RTDR, renal tubular degeneration and regeneration; RTD, renal tubular dilation. Renal score definition: 0, absent; 1, <5% of tubules affected; 2, 5%–25% of tubules affected; 3, 25%–50% of tubules affected; 4, >50% of tubules affected. HMNH, heart myocardial necrosis and hemorrhage: 0, no lesions; 1, <5% of myocardium affected; 2, 5-10% affected; 3, 10-25% affected; 4, >25% affected. HT, heart telangiectasia: 0, absent; 1, present. HPMC, heart perivascular myxomatous change: 0, absent; 1, present. BMH, brain multifocal hemorrhage: 0, absent; 1, present. RES, reticuloendothelial system. KCHMV, Kupffer cell hyperplasia and microvesicular vacuolation: 0, absent; 1, minimal accumulation; 2, mild accumulation; 3, moderate accumulation; 4, severe accumulation. SRPHMV, splenic red pulp histiocytosis with microvesicular vacuolation: 0, no lesions; 1, lesions present. In addition, diffuse hepatic subcellular degeneration and neutrophilic infiltration were seen in one mouse each after 3 and 5 days of 20 mg P-407.

Captions for Supplementary Videos

Supplementary Video 1 | Bioluminescence imaging of Antares luciferase in CaMKIIα-positive neurons in a freely moving mouse. After i.p. injection of CFz, a mouse expressing Antares under the control of a *CaMKIIα-cre driver* was monitored during free movement using an electron-multiplying CCD (EM-CCD) camera. The head was shaved but no surgery was performed; bioluminescence was acquired through intact skull and skin. Bright-field and luminescent images (60 ms exposure time for each) were alternately acquired. The video was recorded at a resolution of 512 × 512 pixels at 8.33 frames per second (fps) for 3 min. A 1-min segment played back at 17 fps (~2× actual speed) is shown. Left, bright field; Right luminescence. Related to **Fig. 4a**.

Supplementary Video 2 | Non-invasive bioluminescence brain imaging of Antares luciferase in Vgat-positive neurons. After retroorbital injection of CFz, a mouse expressing Antares under the control of a *Vgat-cre driver* was imaged during head fixation. The head was shaved but no surgery was performed; bioluminescence was acquired through intact skull and skin. Images were acquired at 5 fps of 20 repeats of 1-s vibrational stimulation to one foot followed by 19 s rest (**Fig. 4b**) and then the 20 repeats were averaged. Left, raw bioluminescence image in gray scale; Right, pseudocolor. Related to **Fig. 4c,e**.

Supplementary Video 3 | Non-invasive bioluminescence brain imaging of CaMBI110 in Vgat-positive neurons. After retroorbital injection of CFz, a mouse expressing CaMBI110 under the control of a *Vgat-cre driver* was imaged during head fixation. The head was shaved but no surgery was performed; bioluminescence was acquired through intact skull and skin. Images were acquired at 5 fps of 20 repeats of 1-s vibrational stimulation to one foot followed by 19 s rest (**Fig. 4b**) and then the 20 repeats were averaged. Left, raw bioluminescence image gray scale; Right, pseudocolor. Related to **Fig. 4d,f**.

Supplementary Note

Scheme 1

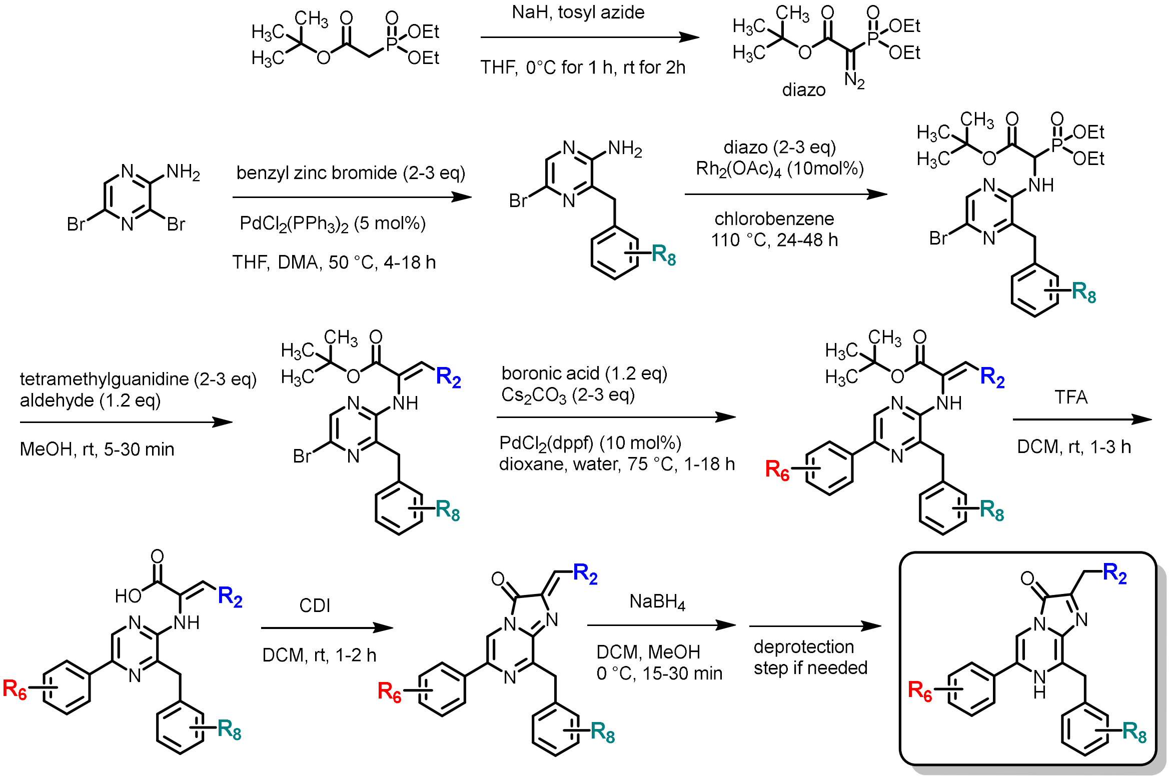

8-benzyl-6-(2-fluorophenyl)-2-(furan-2-ylmethyl)imidazo[1,2-a]pyrazin-3(7H)-one (**4**)

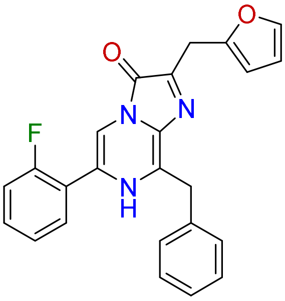

^{1}H NMR (400 MHz, Methanol-d4) δ 7.68 (s, 2H), 7.59 – 7.49 (m, 1H), 7.46 – 7.17 (m, 10H), 6.37 – 6.31 (m, 1H), 6.13 (d, *J* = 3.2 Hz, 1H), 4.42 (s, 2H), 4.22 (s, 2H); ^{13}C NMR (101 MHz, Methanol-d4) δ 161.32, 158.86, 151.54, 141.37, 136.44, 131.43, 130.31, 130.29, 128.64, 128.46, 128.35, 128.14, 126.81, 126.48, 124.61, 124.56, 115.91, 115.68, 110.03, 106.12; HRMS (ESI+) calcd for $C_{24}H_{18}FN_3O_2$ [M + H]+ m/z 400.1462 found 400.1425; HPLC 68.2% (AUC at 254 nm) 2.50 min (Accucore C8, 50x2.1mm, 2.6µm, water/ACN 0.1%TFA).

8-benzyl-6-(3-fluorophenyl)-2-(furan-2-ylmethyl)imidazo[1,2-a]pyrazin-3(7H)-one (**5**)

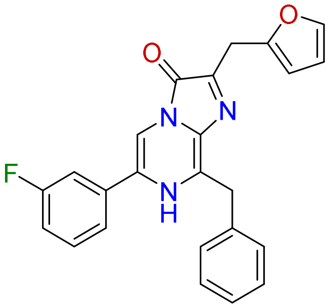

^{1}H NMR (400 MHz, Methanol-d$_4$) δ 7.91 (s, 1H), 7.65 – 7.30 (m, 6H), 7.39 – 7.24 (m, 3H), 7.27 – 7.13 (m, 2H), 6.33 (d, *J* = 3.2, Hz, 1H), 6.12 (*d*, J = 3.2 Hz, 1H), 4.44 (s, 2H), 4.20 (s, 2H); ^{13}C NMR (101 MHz, Methanol-d$_4$) δ 164.29, 161.86, 151.55, 141.40, 136.63, 130.59, 130.51, 128.50, 128.31, 126.73, 122.12, 115.76, 115.56, 113.35, 113.12, 110.04, 108.66, 106.12; HRMS (ESI+) calcd for $C_{24}H_{18}FN_3O_2$ [M + H]+ m/z 400.1462, found 400.1428; HPLC 92.4% (AUC at 254 nm) 2.93 min (Accucore C8, 50x2.1mm, 2.6μm, water/ACN 0.1%TFA).

8-benzyl-6-(2,3-difluorophenyl)-2-(furan-2-ylmethyl)imidazo[1,2-a]pyrazin-3(7H)-one (**6**, **CFz**)

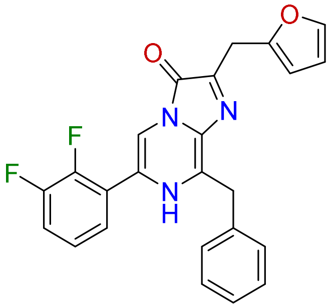

^{1}H NMR (400 MHz, Methanol-d$_4$) δ 7.89 (s, 1H), 7.54 (s, 1H), 7.46 – 7.37 (m, 4H), 7.41 – 7.21 (m, 5H), 6.37 – 6.31 (m, 1H), 6.12 (d, *J* = 3.2 Hz, 1H), 4.43 (s, 2H), 4.22 (s, 2H); ^{13}C NMR (101 MHz, Methanol-d$_4$) δ 151.56, 149.59, 149.54, 141.39, 136.49, 128.50, 128.32, 126.77, 125.22, 125.19, 124.78, 124.74, 124.71, 124.67, 118.10, 117.91, 111.13, 110.01, 106.11; HRMS (ESI+) calcd for $C_{24}H_{17}F_2N_3O_2$ [M + H]+ m/z 418.1368, found 418.1330; HPLC 99.0% (AUC at 254 nm) 2.94 min (Accucore C8, 50x2.1mm, 2.6μm, water/ACN 0.1%TFA).

8-(2-fluorobenzyl)-6-(3-fluorophenyl)-2-(furan-2-ylmethyl)imidazo[1,2-a]pyrazin-3(7H)-one (**7**)

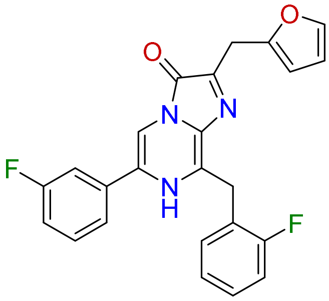

^{1}H NMR (400 MHz, Methanol-d$_4$) δ 8.17 (s, 1H), 7.61 (d, *J* = 7.8 Hz, 1H), 7.54 (d, *J* = 10.4 Hz, 1H), 7.45 (td, *J* = 8.0, 5.9 Hz, 1H), 7.40 (s, 1H), 7.38 – 7.22 (m, 2H), 7.20 – 7.05 (m, 3H), 6.33 (d, *J* = 3.2 Hz, 1H), 6.12 (d, *J* = 3.2 Hz, 1H), 4.51 (s, 2H), 4.19 (s, 2H); ^{13}C NMR (101 MHz, Methanol-d$_4$) δ 164.35, 162.33, 161.91, 159.89, 151.59, 141.42, 130.72, 130.45, 130.36, 128.73, 128.64, 124.04, 123.99, 123.81, 123.65, 121.71, 115.49, 115.29, 114.94, 114.71, 113.07, 112.83, 110.03, 108.99, 106.06; HRMS (ESI+) calcd for $C_{24}H_{17}F_2N_3O_2$ [M + H]+ m/z 418.1368, found 418.1329; HPLC 95.7% (AUC at 254 nm) 3.08 min (Accucore C8, 50x2.1mm, 2.6μm, water/ACN 0.1%TFA).

8-(2-fluorobenzyl)-6-(2-fluorophenyl)-2-(furan-2-ylmethyl)imidazo[1,2-a]pyrazin-3(7H)-one (**8**)

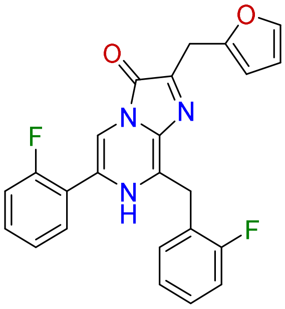

^{1}H NMR (400 MHz, Methanol-d$_4$) δ 7.98 (s, 1H), 7.75 (s, 1H), 7.51 – 7.45 (m, 1H), 7.40 (t, *J* = 2.8 Hz, 1H), 7.35 – 7.20 (m, 4H), 7.18 – 7.04 (m, 2H), 6.36 – 6.28 (m, 1H), 6.12 (d, *J* = 3.2 Hz, 1H), 4.50 (s, 2H), 4.20 (s, 2H); ^{13}C NMR (101 MHz, Methanol-d$_4$) δ 162.26, 161.36, 159.80, 158.89, 151.55, 141.38, 130.97, 130.50, 160.19, 160.16, 128.80, 128.72., 124.52, 124.46, 124.09, 124.04, 115.87, 115.65, 115.00, 114.75, 110.03, 106.09; HRMS (ESI+) calcd for $C_{24}H_{17}F_2N_3O_2$ [M + H]+ m/z 418.1368, found 418.1329; HPLC 92.1% (AUC at 254 nm) 2.88 min (Accucore C8, 50x2.1mm, 2.6μm, water/ACN 0.1%TFA).

6-(2,3-difluorophenyl)-8-(2-fluorobenzyl)-2-(furan-2-ylmethyl)imidazo[1,2-a]pyrazin-3(7H)-one (**9**)

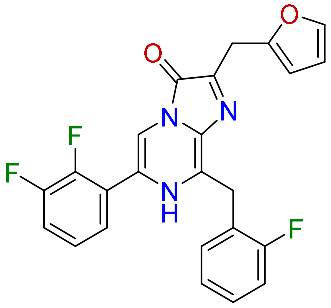

^{1}H NMR (400 MHz, Methanol-d$_4$) δ 8.12 (s, 1H), 7.57 (t, *J* = 7.3 Hz, 1H), 7.42 – 7.18 (m, 5H), 7.20 – 7.10 (m, 2H), 6.33 (dd, *J* = 3.2, 1.9 Hz, 1H), 6.11 (d, *J* = 3.2 Hz, 1H), 4.50 (s, 2H), 4.20 (s, 2H); ^{13}C NMR (101 MHz, Methanol-d$_4$) δ 162.32, 159.87, 152.03, 151.90, 151.65, 149.59, 149.46, 147.10, 146.96, 141.39, 130.74, 130.70, 128.74, 128.66, 126.64, 125.05, 125.02, 124.56, 124.51, 124.48, 124.43, 124.03, 123.99, 123.69, 123.53, 117.63, 117.47, 114.94, 114.72, 111.88, 110.01, 106.06; HRMS (ESI+) calcd for $C_{24}H_{16}F_3N_3O_2$ [M + H]+ m/z 436.1274, found 436.1236; HPLC 96.9% (AUC at 254 nm) 3.08 min (Accucore C8, 50x2.1mm, 2.6µm, water/ACN 0.1%TFA).

8-benzyl-6-(2,3-difluorophenyl)-2-((5-methylfuran-2-yl)methyl)imidazo[1,2-a]pyrazin-3(7H)-one (**10**)

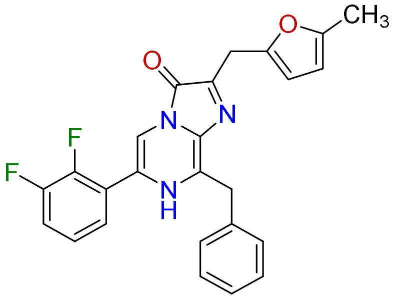

^{1}H NMR (400 MHz, Methanol-d$_4$) δ 7.89 (s, 1H), 7.53 (s, 1H), 7.46 – 7.20 (m, 8H), 5.97 (d, *J* = 3.0 Hz, 1H), 5.90 (d, *J* = 3.0 Hz, 1H), 4.42 (s, 2H), 4.15 (s, 2H), 2.23 (s, 3H); ^{13}C NMR (101 MHz, Methanol-d$_4$) δ 151.99, 151.86, 150.83, 149.51, 149.41, 147.08, 146.92, 136.50, 128.51, 128.31, 126.76, 125.20, 125.16, 124.76, 124.69, 124.63, 118.85, 117.89, 111.14, 106.85, 105.84, 12.03; HRMS (ESI+) calcd for $C_{25}H_{19}F_2N_3O_2$ [M + H]+ m/z 432.1524, found 432.1493; HPLC 98.3% (AUC at 254 nm) 3.11 min (Accucore C8, 50x2.1mm, 2.6µm, water/ACN 0.1%TFA).

6-(2,3-difluorophenyl)-8-(2-fluorobenzyl)-2-(thiophen-2-ylmethyl)imidazo[1,2-a]pyrazin-3(7H)-one (**11**)

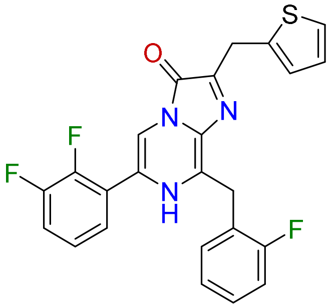

^{1}H NMR (400 MHz, Methanol-d$_4$) δ 8.07 (s, 1H), 7.56 (s, 1H), 7.41 – 7.17 (m, 4H), 7.17 – 7.04 (m, 2H), 6.98 – 6.86 (m, 2H), 4.50 (s, 2H), 4.36 (s, 2H); ^{13}C NMR (101 MHz, Methanol-d$_4$) δ 162.26, 159.84, 152.03, 151.91, 149.60, 149.46, 147.09, 146.97, 141.05, 140.66, 130.98, 130.70, 128.80, 128.70, 126.39, 125.12, 125.07, 125.03, 124.97, 124.53, 124.06, 124.02, 123.77, 114.96, 114.76; HRMS (ESI+) calcd for $C_{24}H_{16}F_3N_3OS$ [M + H]+ m/z 452.1045, found 452.1009; HPLC 88.2% (AUC at 254 nm) 3.25 min (Accucore C8, 50x2.1mm, 2.6µm, water/ACN 0.1%TFA).

6-(2,3-difluorophenyl)-8-(2-fluorobenzyl)-2-(4-fluorobenzyl)imidazo[1,2-a]pyrazin-3(7H)-one (**12**)

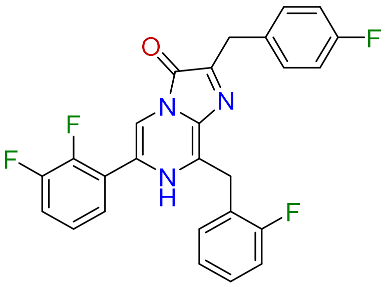

^1H NMR (400 MHz, Methanol-d_4) δ 8.07 (s, 1H), 7.56 (s, 1H), 7.42 – 7.20 (m, 6H), 7.18 – 7.08 (m, 2H), 7.02 (t, *J* = 8.8 Hz, 2H), 4.49 (s, 2H), 4.17 (s, 2H); ^{13}C NMR (101 MHz, Methanol-d_4) δ 162.86, 162.27, 160.45, 159.84, 152.03, 151.90, 149.59, 149.44, 134.48, 130.61, 130.05, 129.97, 128.79, 128.71, 125.05, 125.00, 124.53, 124.06, 124.01, 123.62, 123.47, 117.68, 114.96, 114.78, 114.74, 114.53; HRMS (ESI+) calcd for $C_{26}H_{17}F_4N_3O$ [M + H]+ m/z 464.1387, found 464.1355; HPLC 99.1% (AUC at 254 nm) 3.40 min (Accucore C8, 50x2.1mm, 2.6µm, water/ACN 0.1%TFA).

6-(2,3-difluorophenyl)-8-(2-fluorobenzyl)-2-((5-methylfuran-2-yl)methyl)imidazo[1,2-a]pyrazin-3(7H)-one (**13**)

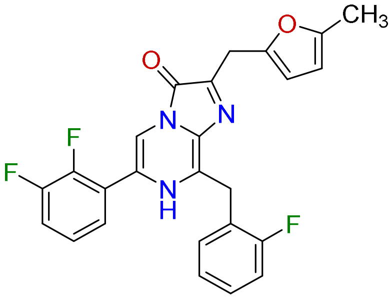

^1H NMR (400 MHz, Methanol-d_4) δ 8.11 (s, 1H), 7.60 – 7.49 (m, 1H), 7.38 – 7.23 (m, 3H), 7.27 – 7.17 (m, 1H), 7.15 – 7.05 (m, 2H), 5.96 (d, *J* = 3.0 Hz, 1H), 5.88 (d, *J* = 3.0 Hz, 1H), 4.50 (s, 2H), 4.14 (s, 2H), 2.22 (s, 3H); ^{13}C NMR (101 MHz, Methanol-d_4) δ 162.31, 159.87, 152.04, 151.91, 150.86, 149.55, 149.45, 147.11, 146.96, 130.72, 128.76, 128.68, 125.04, 125.01, 124.56, 124.49, 124.43, 124.03, 124.00, 123.67, 123.51, 117.65, 117.47, 114.95, 114.73, 111.84, 106.81, 105.84, 12.02; HRMS (ESI+) calcd for $C_{25}H_{18}F_3N_3O_2$ [M + H]+ m/z 450.1430, found 450.1383; HPLC 98.8% (AUC at 254 nm) 3.29 min (Accucore C8, 50x2.1mm, 2.6µm, water/ACN 0.1%TFA).

HRMS spectrum for compound **4**

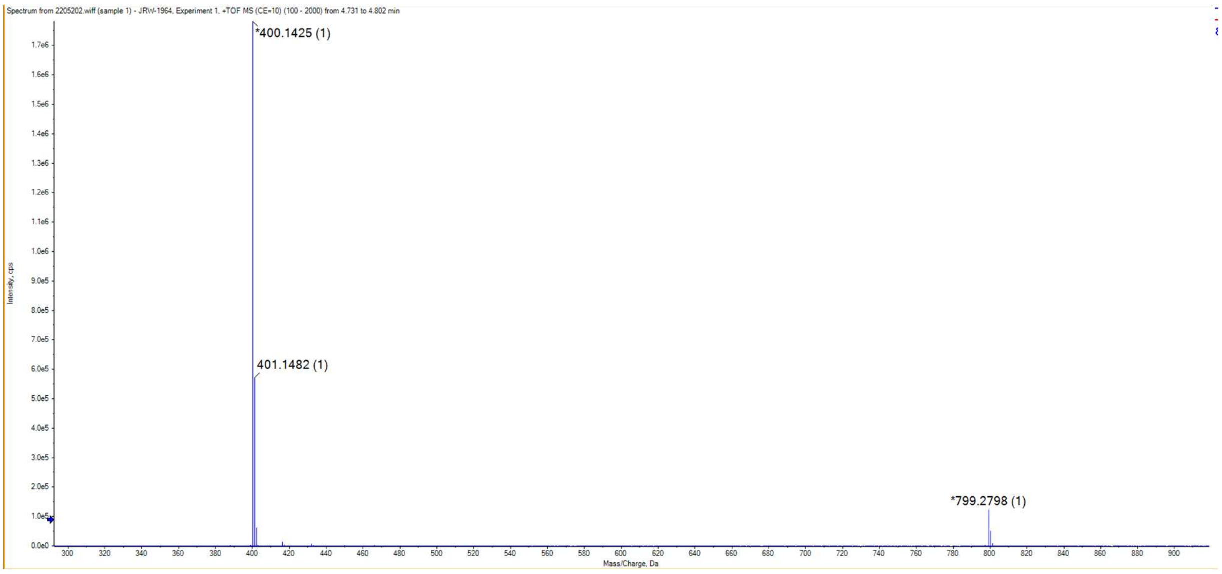

HRMS spectrum for compound **5**

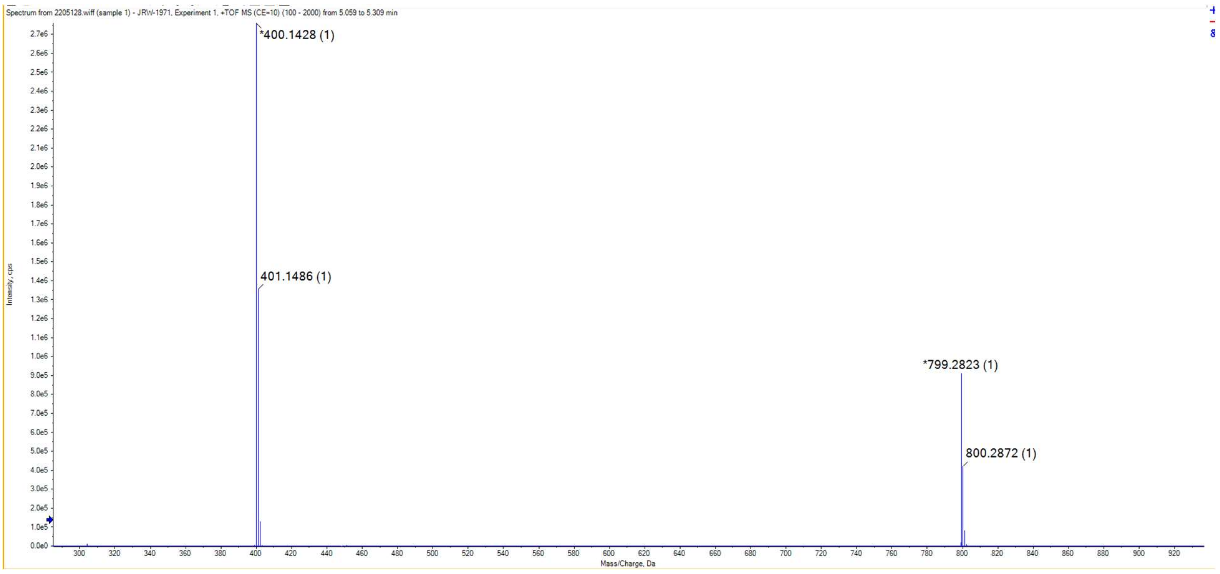

HRMS spectrum for compound **6**

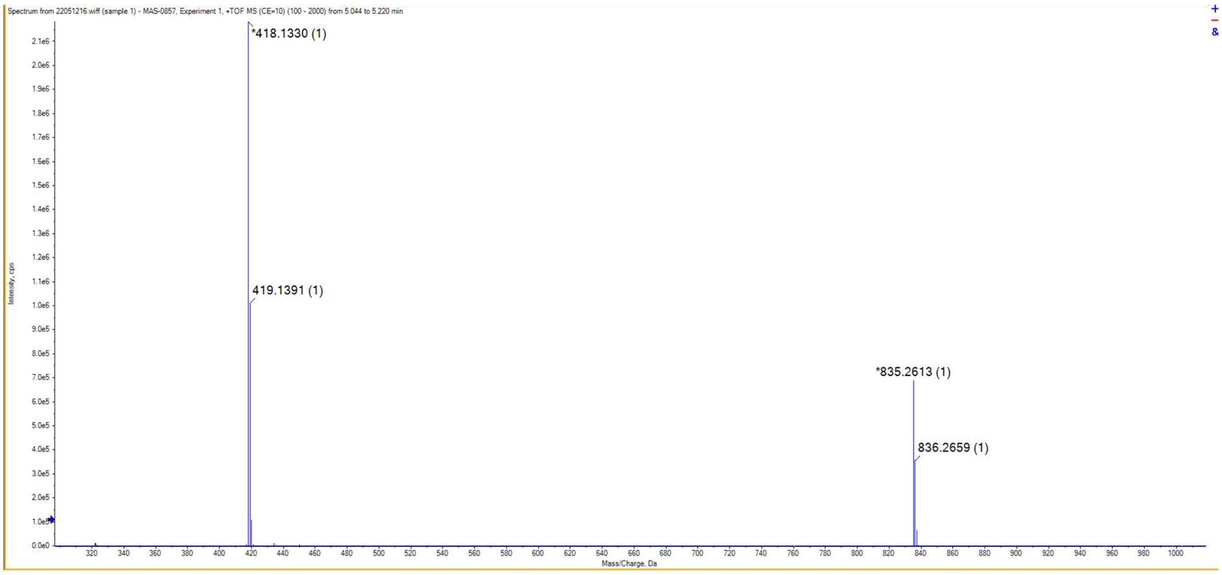

HRMS spectrum for compound **7**

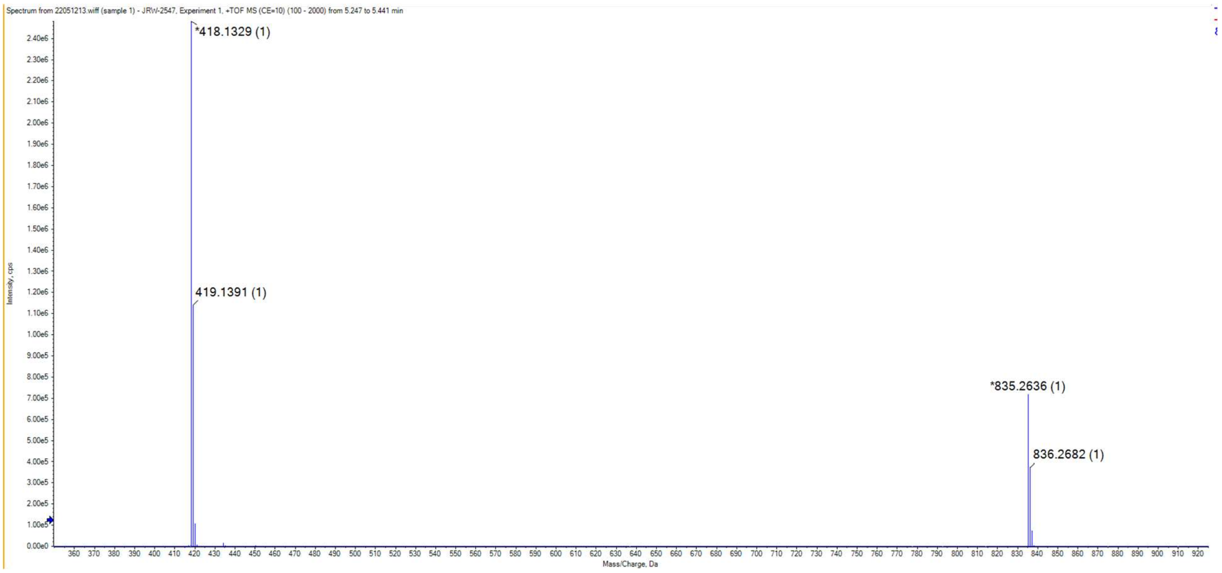

HRMS spectrum for compound **8**

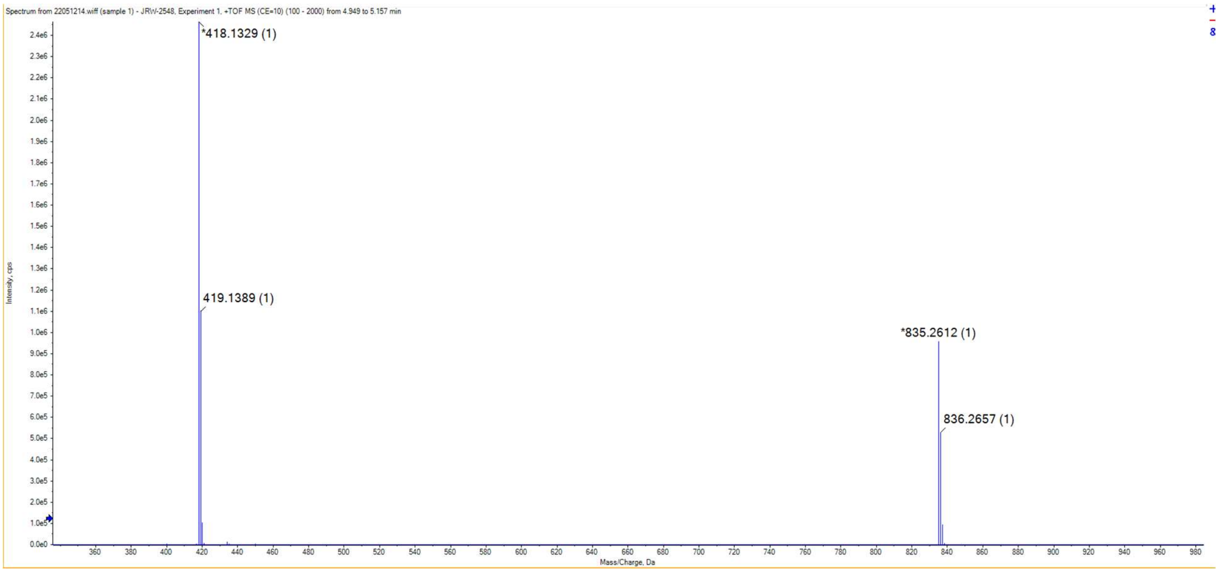

HRMS spectrum for compound **9**

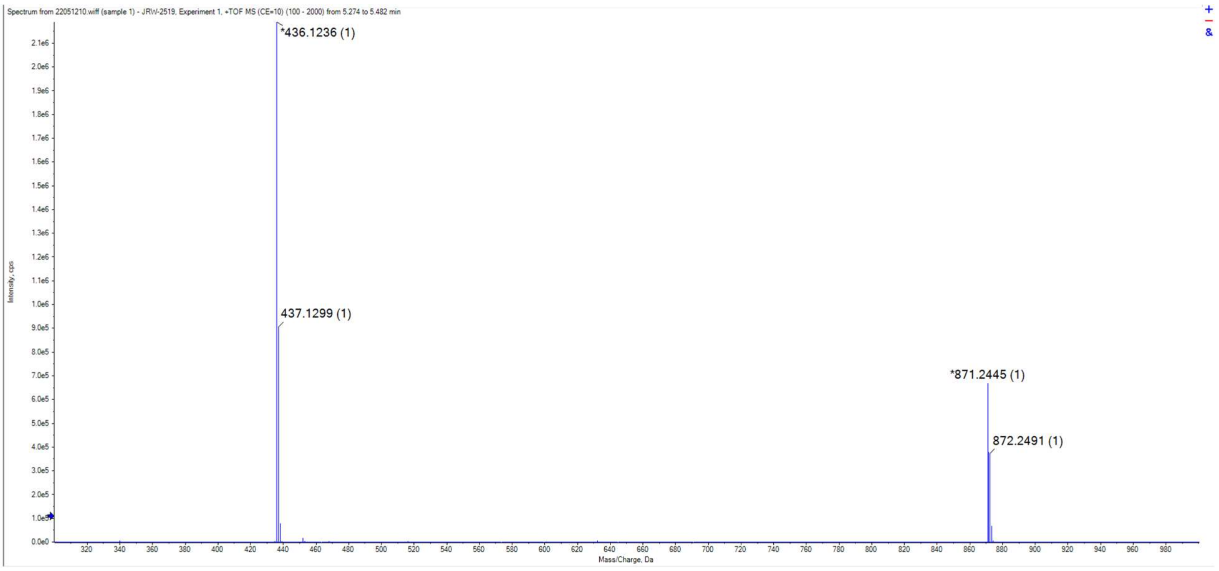

HRMS spectrum for compound **10**

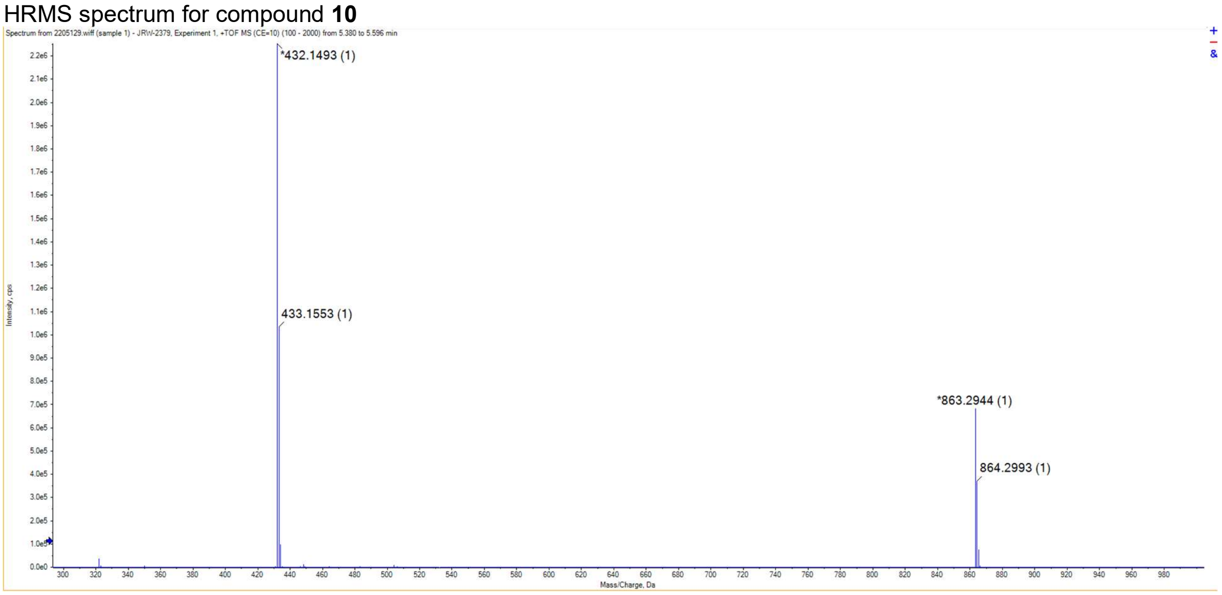

HRMS spectrum for compound **11**

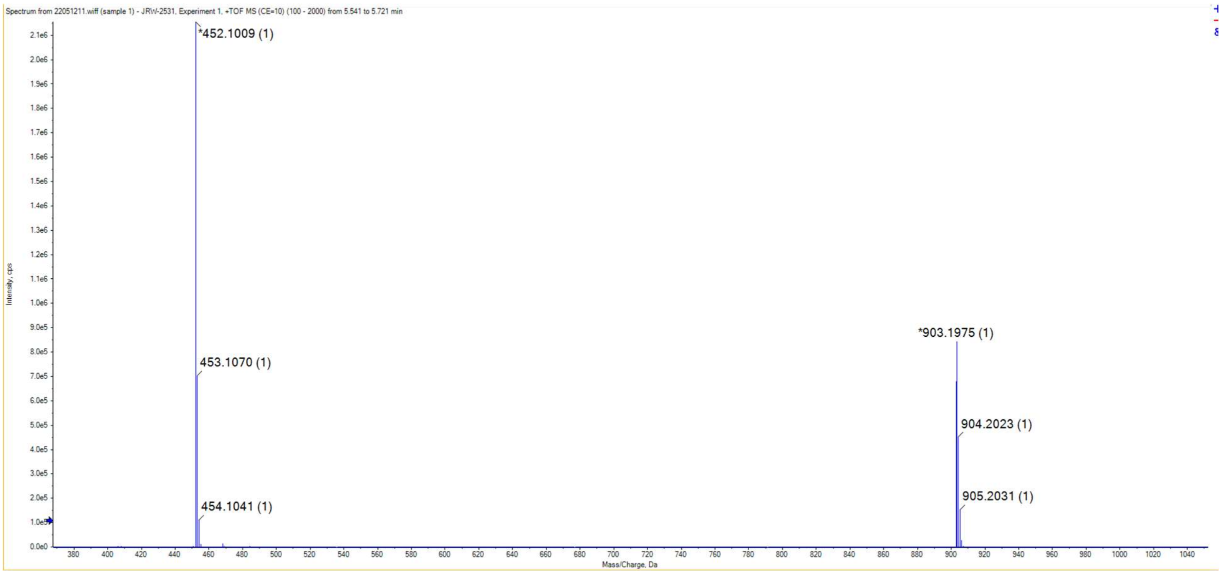

HRMS spectrum for compound **12**

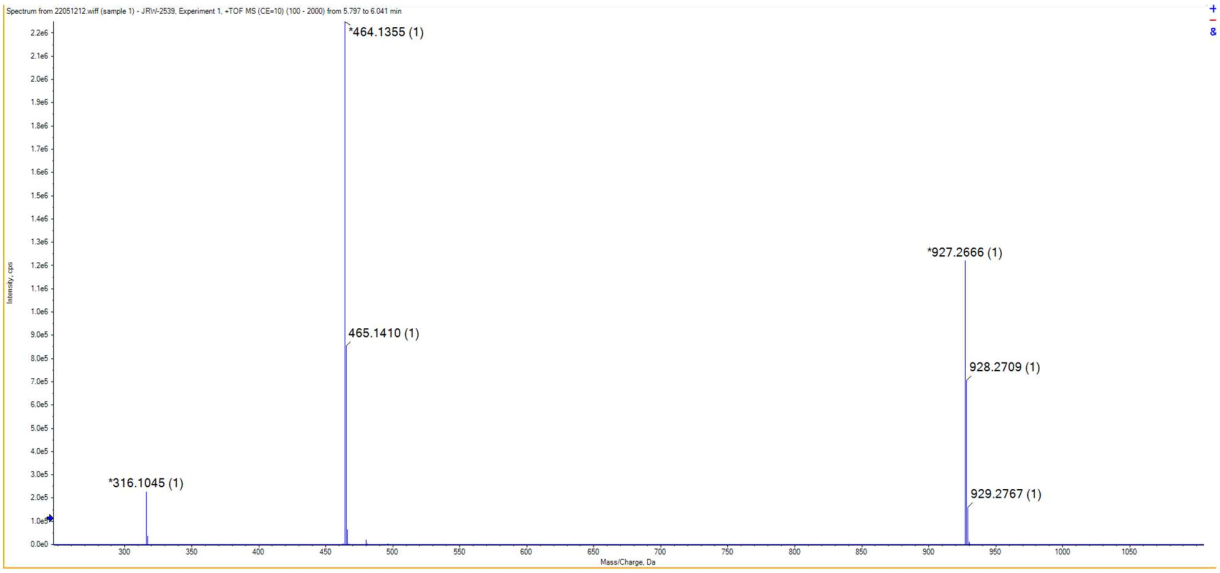

HRMS spectrum for compound **13**

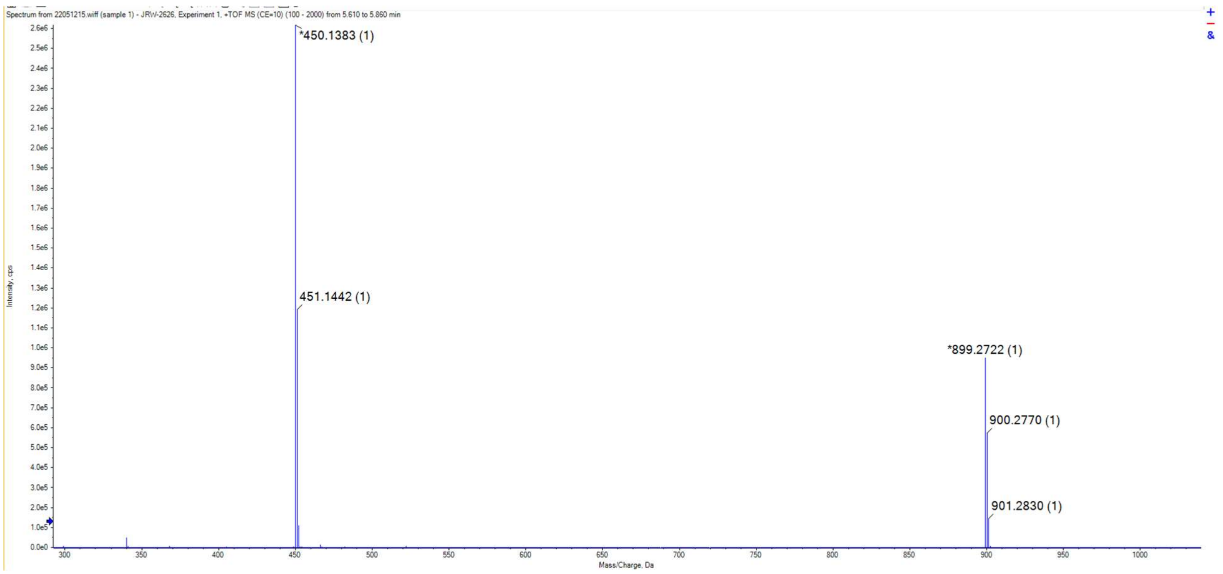

Supplement: Supplementary file 1 — Supplementary Tables 1–3. [file 41589_2023_1265_MOESM1_ESM.pdf]
