# Supplementary material for: An optimized bioluminescent substrate for non-invasive imaging in the brain
Source: Nat Chem Biol. 2023 Feb 9;19(6):731–9. doi: 10.1038/s41589-023-01265-x (PMC10229426; doi:10.1038/s41589-023-01265-x)
Supplement: Supplementary file 2 — Reporting Summary [file 41589_2023_1265_MOESM2_ESM.pdf]

nature research

Corresponding author(s): Dr. Michael Z. Lin, Dr. Thomas A. Kirkland

Last updated by author(s): Dec 26, 2022

Reporting Summary

Nature Research wishes to improve the reproducibility of the work that we publish. This form provides structure for consistency and transparency in reporting. For further information on Nature Research policies, see our Editorial Policies and the Editorial Policy Checklist.

Statistics

For all statistical analyses, confirm that the following items are present in the figure legend, table legend, main text, or Methods section.

n/a	Confirmed	
☐	☒	The exact sample size (*n*) for each experimental group/condition, given as a discrete number and unit of measurement
☐	☒	A statement on whether measurements were taken from distinct samples or whether the same sample was measured repeatedly
☐	☒	The statistical test(s) used AND whether they are one- or two-sided<br>*Only common tests should be described solely by name; describe more complex techniques in the Methods section.*
☒	☐	A description of all covariates tested
☐	☒	A description of any assumptions or corrections, such as tests of normality and adjustment for multiple comparisons
☐	☒	A full description of the statistical parameters including central tendency (e.g. means) or other basic estimates (e.g. regression coefficient) AND variation (e.g. standard deviation) or associated estimates of uncertainty (e.g. confidence intervals)
☐	☒	For null hypothesis testing, the test statistic (e.g. *F*, *t*, *r*) with confidence intervals, effect sizes, degrees of freedom and *P* value noted<br>*Give P values as exact values whenever suitable.*
☒	☐	For Bayesian analysis, information on the choice of priors and Markov chain Monte Carlo settings
☒	☐	For hierarchical and complex designs, identification of the appropriate level for tests and full reporting of outcomes
☒	☐	Estimates of effect sizes (e.g. Cohen's *d*, Pearson's *r*), indicating how they were calculated

Our web collection on statistics for biologists contains articles on many of the points above.

Software and code

Policy information about availability of computer code

Data collection	Aura imaging software, version: 3.1 or 4.0; Andor Solis software, version: 4.32
Data analysis	ImageJ, version:2.1.0; Microsoft Excel, version:16.54; GraphPad Prism, version 9.0.0; Ami Aura imaging software, version 3.1 or 4.0; Matlab, version: 9.11.0; Pharsight Phoenix WinNonlin® 8.2 software

For manuscripts utilizing custom algorithms or software that are central to the research but not yet described in published literature, software must be made available to editors and reviewers. We strongly encourage code deposition in a community repository (e.g. GitHub). See the Nature Research guidelines for submitting code & software for further information.

Data

Policy information about availability of data

All manuscripts must include a data availability statement. This statement should provide the following information, where applicable:

- Accession codes, unique identifiers, or web links for publicly available datasets
- A list of figures that have associated raw data
- A description of any restrictions on data availability

The main data supporting the findings of this study are available within the Article and its Supplementary Information. The raw data generated in this study are available from the corresponding author upon request.

Field-specific reporting

Please select the one below that is the best fit for your research. If you are not sure, read the appropriate sections before making your selection.

☒ Life sciences ☐ Behavioural & social sciences ☐ Ecological, evolutionary & environmental sciences

For a reference copy of the document with all sections, see nature.com/documents/nr-reporting-summary-flat.pdf

Life sciences study design

All studies must disclose on these points even when the disclosure is negative.

Sample size

Using standard deviation values measured in preliminary experiments, a power calculation using G*Power software revealed a 80% likelihood of detecting a 50% difference in brain signals, measured relative to the lower value, with 3 animals. We thus aimed for 3 animals per condition, but if more animals were available from breeding, they were included as well.

Data exclusions

In the toxicity study (Extended Data Fig. 5-7, Supplementary Table 1), one set of mice receiving 3 daily doses of 1.3 µmol CFz was revealed heart damage that appeared to have originated pre-test. We repeated 3 daily doses of 1.3 µmole CFz on a new set of mice.
There are no data exclusions involved in the other experiments described in the study.

Replication

Experiments for luciferase substartes comparison, toxicity study and in vivo pharmacokinetics study were done with only one single independent repeat, but with sufficient biological replicates (see "Sample size" above) for statistical analysis of the comparison results. The number of biological replicates for individual experiments were stated in each figure captions.
Experiments for brain activity imaging (Ex Fig 9a, Ex Fig 10) were repeated at least twice on the same mouse or mice with same sex and same age with similar results.

Randomization

All experimental conditions used a common set of starting materials and were performed in parallel.
For comparing different substrates in vivo (Fig. 1c-d, Fig. 2e, Fig. 3a-d (for AkaLuc and FLuc), Fig. 3e-f, Ex Fig 2b, Ex Fig. 3, Ex Fig. 4, Ex Fig 8a-b (for Fluc), Ex Fig 8c-d (for FFz vs CFz)), the comparison of each set of substrates was done on the same group of mice, with multiple injections on different days. The order for each mouse receiving the different substrate was designed randomly.
For some other experiments comparing different substrates in vivo (Ex Fig. 2a,c, Ex Fig 7c), in which more animals were available from breeding, the experiment was done on the mice of the same sex and similar age (difference < 1 week) that were divided randomly into groups to receive different substrates, so that each mouse will only receive one injection of substrate for humane reason.
For in vivo PK measurements (Fig 2c,d) and toxicity study (Ex Fig. 5-7, Supplementary Table 1), C57BL/6J mice of the same sex (male)and similar age (difference < 1 week) were randomly divided into groups to receive different substrates.

Blinding

For experiments of comparing luciferase substrates, the experimentor designed the experimental conditions and operated the injections, but the results were directly provided by the in vivo imager. There were no subjective judgment involved in the data acquisition and analysis steps so there was no blinding used.
For the toxicity test (Ex Fig. 5-7, Supplementary Table 1), one experimentor numbered and grouped the mice and performed the injection of substrates, while the other experimentors blindly performed the dissection, carried out histology analysis and scaled the organ damaged.
For analyzing the calcium imaging data (Fig. 4b-e, Ex Fig. 9, Ex Fig. 10), the same matlab code and image processing were first blindingly applied on the mice without knowing the identities and conditions.

Reporting for specific materials, systems and methods

We require information from authors about some types of materials, experimental systems and methods used in many studies. Here, indicate whether each material, system or method listed is relevant to your study. If you are not sure if a list item applies to your research, read the appropriate section before selecting a response.

Materials & experimental systems

n/a	Involved in the study
☒	☐ Antibodies
☒	☐ Eukaryotic cell lines
☒	☐ Palaeontology and archaeology
☐	☒ Animals and other organisms
☒	☐ Human research participants
☒	☐ Clinical data
☒	☐ Dual use research of concern

Methods

n/a	Involved in the study
☒	☐ ChIP-seq
☒	☐ Flow cytometry
☒	☐ MRI-based neuroimaging

Animals and other organisms

Policy information about studies involving animals; ARRIVE guidelines recommended for reporting animal research

Laboratory animals

Housing conditions: mice were maintained under 20-24 Celsius degree, 45-65% humidity and 12-12 hour dark-light cycles.
C57BL/6J mice, male, 6-16 weeks old;

	J:NU mice (#7850 EC, Jackson Laboratories), male, 7-8 weeks old;<br>LSL-CAG-reporter transgenic mice (C57BL/6J background, generated by Stanford TKTC, reporters: Antares, CaMBI110, FLuc or AkaLuc), male and female, 6-16 weeks old, for breeding;<br>CamkIIa-cre (Jackson laboratory, No. 005359), male and female, 6-16 weeks old, for breeding.<br>Vgat-ires-cre (Jackson laboratory, No. 28862), male and female, 6-16 weeks old, for breeding.<br>Vglut2-ires-cre (Jackson laboratory, No. 016963), male and female, 6-16 weeks old, for breeding.<br>Mice hemizygous for LSL-CAG-reporter and cre-driver, male and female, 6-12 weeks old, for in vivo imaging.
Wild animals	This study doesn't involve wild animals
Field-collected samples	This study doesn't involve field-collected samples
Ethics oversight	The animal study is following a protocol approved by Administrative Panel on Laboratory Animal Care (APLAC), the IACUC at Stanford University

Note that full information on the approval of the study protocol must also be provided in the manuscript.
